# An extension of jamdock-suite for virtual screening from raw chemical datasets

**DOI:** 10.1016/j.xpro.2026.104822

**Published:** 2026-09-07

**Authors:** Shahariar Emon, Md. Mozzammel Haque, Iftekhar Alam, Md. Anwarul Haque, Jahangir Md. Alam

**Affiliations:** 1 Department of Biotechnology and Genetic Engineering, Islamic University, Kushtia 7003, Bangladesh; 2 Department of Urology, Satkhira Medical College, Satkhira 9400, Bangladesh; 3 Plant Biotechnology Division, National Institute of Biotechnology, Ganakbari, Ashulia, Savar, Dhaka 1349, Bangladesh

**Keywords:** Bioinformatics, Biophysics, High Throughput Screening

## Abstract

Virtual screening of chemical libraries has long been a cornerstone for identifying candidate bioactive compounds. Recently, many tools have been developed to make virtual screening more accessible. Among them, the jamdock-suite provides an automated and user-friendly workflow that encompasses the entire process from library generation to docking evaluation. However, its library generation is restricted to the ZINC database and cannot process user-supplied chemical datasets. Here, we present semdock, an extension of the original workflow that enables the preparation of docking-ready ligands from raw SDF, SMILES, and CSV datasets through an automated ligand preparation procedure. The workflow was validated using 3,230 FDA-approved compounds and multiple protein targets. We also implemented ligand-wise CPU-parallel docking, reducing runtime by approximately 30% compared with conventional multithreaded execution. Evaluation across multiple protein targets confirmed that the extended workflow preserves the utility of the original framework while expanding its use to diverse public and user-defined datasets.

## Highlights

• Virtual screening from raw SDF, SMILES, and CSV chemical datasets
• Automated ligand preparation from raw structures to docking-ready PDBQT files
• CPU-parallel docking using one ligand per CPU core
• Threshold-based filtering and organization of virtual screening results

## Graphical abstract

**Figure:**
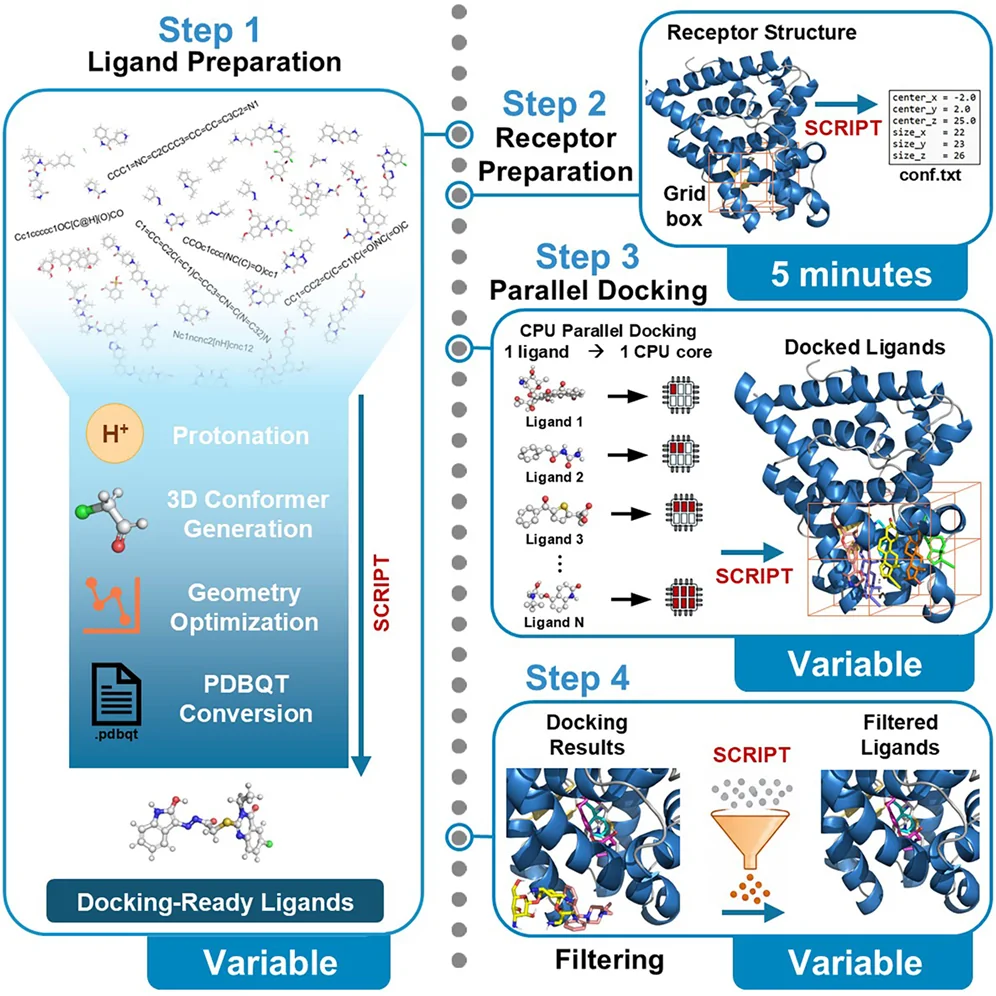

## Introduction

Virtual screening using molecular docking simulations has been a key strategy to evaluate large-scale compound libraries and prioritize promising candidates.^1^^,^^2^^,^^3^ Molecular docking simulations are used to study how proteins and ligands interact. The process starts with a target protein of known structure. Docking then predicts the binding affinity of small molecules to that target. Recent advances in virtual chemical libraries offer millions to billions of synthesizable compounds. This creates a growing need to prioritize candidates for experimental testing. Publicly accessible resources such as PubChem^4^ and ZINC^5^^,^^6^ offer large collections of compounds that can be screened against biological targets for identifying novel chemotypes and drug candidates. Furthermore, the availability of open-source software has expanded the use of virtual screening beyond the pharmaceutical industry. As a result, computational drug discovery is now accessible to academic laboratories and smaller research groups.

Among available docking programs, AutoDock Vina^7^^,^^8^ (Vina) is one of the most widely used open-source tools for molecular docking. However, performing docking simulation with Vina requires specific file formats (PDBQT) of ligand and receptor to process. But, ligand structures available in public compound repositories are distributed as SDF, SMILES, or other chemical structure formats. Preparing docking-ready ligands from these files requires a series of preprocessing steps. Performing these tasks manually for thousands to millions of compounds is laborious and time-consuming. As a result, ligand preparation remains a major practical bottleneck in large-scale virtual screening. Inconsistent preprocessing, skipped steps, and limited automation can reduce efficiency and compromise prediction quality. In addition, downstream analysis and prioritization of large docking datasets introduce additional challenges for users with limited computational experience.

To address these challenges, many tools are greatly contributing to make molecular docking more accessible and automated.^9^^,^^10^^,^^11^^,^^12^^,^^13^ These tools reduce manual intervention and facilitate large-scale screening. However, many still require substantial computational expertise, preprocessed ligand libraries, or complex software configuration. Recently, Barbosa Pereira et al.^14^ introduced jamdock-suite workflow, which integrates automated library generation, receptor preparation, docking and hit identification into a beginner-friendly protocol. The protocol provides an accessible solution for virtual screening with minimal manual interventions.

Despite its utility, jamdock-suite solely depends on ZINC database services for compound library generation, which restricts its use with compounds from other public repositories or user-defined collections. In practice, many screening projects begin with compounds collected from PubChem, ChEMBL, vendor libraries, natural product databases, or in-house collections. Preparing these datasets for docking requires additional preprocessing steps that are not addressed within the original workflow.

Here, we present semdock workflow, an extension of the jamdock-suite workflow that works directly with raw chemical datasets in common formats like SDF, SMILES, and CSV. These formats are widely available in public databases and can be downloaded reliably, even under heavy server load. The workflow automates ligand preparation from given datasets where compounds are standardized, protonated, converted into three-dimensional conformations, geometry-optimized, and transformed into docking-ready PDBQT files. Moreover, the sem_dock module within the semdock workflow performs CPU-parallelized docking using AutoDock Vina to improve computational resource utilization. To evaluate the reliability and computational impact of these modifications, we tested the extended workflow with several protein targets and compound collections, which demonstrates the robustness of ligand preparation, docking performance, and preservation of biologically meaningful docking results. By extending support beyond ZINC-derived libraries, semdock workflow broadens the applicability of automated virtual screening for diverse public and user-generated chemical datasets.

## Results

### Extension of ligand preparation beyond ZINC-derived libraries

The original jamdock-suite^14^ workflow consists of five local scripts: jamlib, jamreceptor, jamqvina, jamresume, and jamrank. In this framework, jamlib generates a compound library from the ZINC database with the inputs from the user. jamreceptor prepares receptor structures, jamqvina performs docking simulations, jamresume enables recovery of interrupted jobs, and jamrank ranks docking results. This framework provides an end-to-end virtual screening workflow with minimal user intervention.

However, the jamlib is fully dependent on external servers from the ZINC database. This works for many screening applications, but it limits the use of compound collections from other sources and creates reliance on the availability of external servers. In practice, researchers often gather compounds from PubChem, ChEMBL, vendor catalogs, natural product databases, or in-house inventories. These datasets are usually shared as SDF, SMILES, or CSV files, so they need extra preparation before docking.

**Figure 1:**
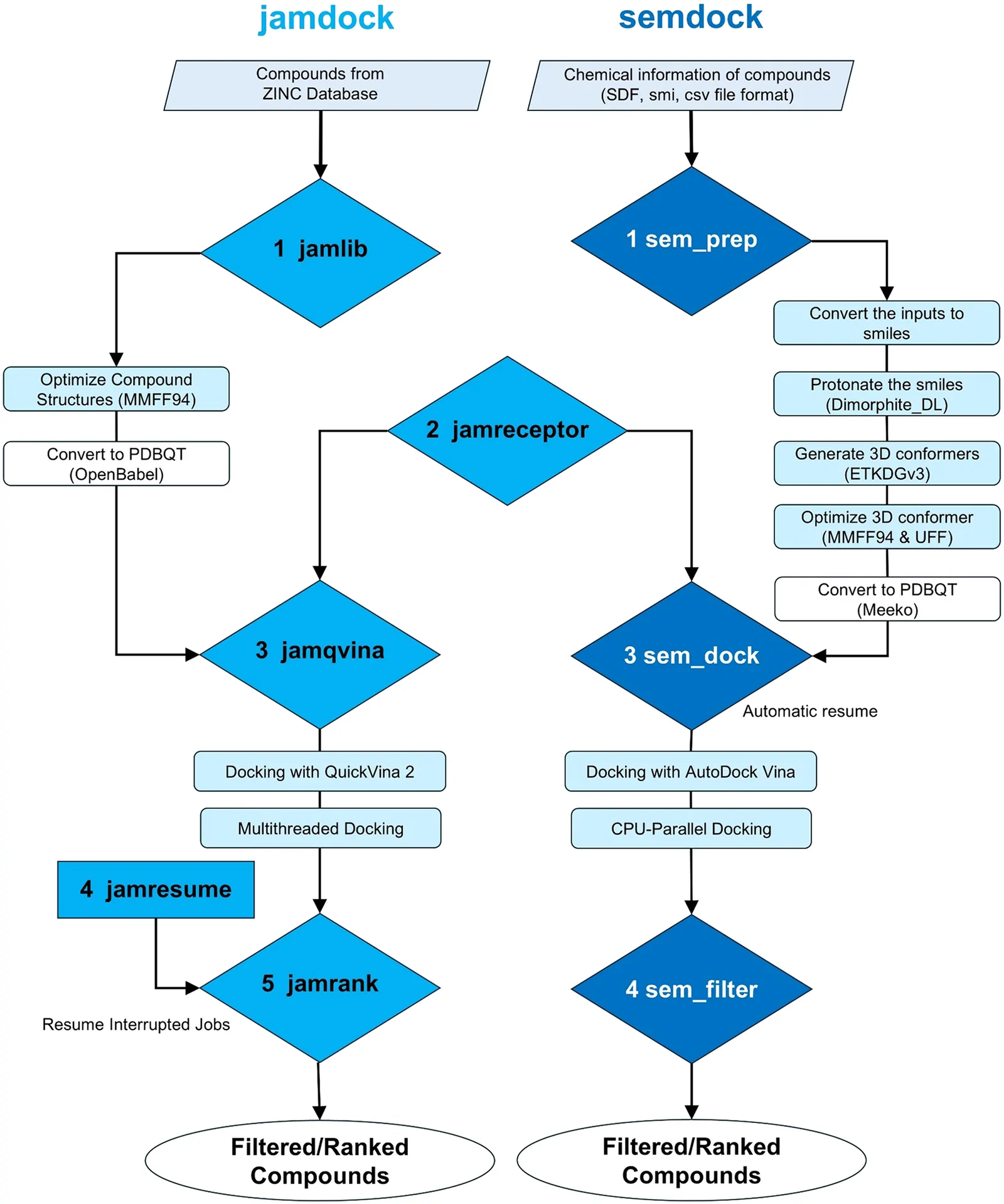
Comparison of the original jamdock-suite and extended semdock workflows semdock extends the original jamdock-suite by enabling direct preparation of docking-ready ligands from raw chemical datasets and introducing ligand-wise CPU-parallel docking and automated post-docking analysis.

To address these limitations, we developed sem_prep, the ligand-preparation module of the extended semdock workflow. It replaces the ZINC-dependent library-generation step with a system that can process raw chemical datasets directly. Instead of retrieving compounds from a fixed source, the sem_prep module takes user-supplied collections and converts them into docking-ready structures. The process is automated and includes standardization, protonation, conformer generation, geometry optimization, and PDBQT conversion. A schematic comparison between the original jamdock-suite workflow and the extended semdock workflow is shown in Figure 1.

These modifications expand the applicability of the original protocol beyond ZINC-derived libraries and allow virtual screening projects to begin directly from heterogeneous chemical datasets. By using established cheminformatics tools, the workflow generates chemically reasonable docking-ready structures from diverse compound collections. At the same time, it keeps the automation and accessibility of the original protocol. As a result, the extended protocol can be used for more applications, including drug repurposing studies, screening curated compound collections, and analyzing user-defined libraries.

To evaluate the robustness of the modified ligand-preparation procedure, we downloaded a library of 3,230 FDA-approved compounds from the ZINC database in CSV format (Data S7). Successful ligand preparation was defined as completion of all processing steps, which include standardization, protonation-state assignment, 3D conformer generation, geometry optimization, and conversion into docking-ready PDBQT format. Out of 3230 compounds, 3,171 were successfully processed, giving a success rate of 98.17%. Only 59 compounds (1.83%) failed during preparation. The FDA-approved compounds include diverse structures like peptides, macrocycles, highly charged, and stereochemically complex drugs. Most compounds were prepared successfully, showing that the module works well for varied molecular types. The few failures were mainly associated with highly rigid structures.

Inspection of failed molecules indicated that most failures occurred during the conformer-generation stage. Compared with successfully prepared ligands, the failed compounds were composed of structurally constrained molecules such as bridges or polycyclic scaffolds, spiro junctions, and multiple stereocenters. These features increase topological rigidity, which makes it difficult for the ETKDGv3 distance-geometry algorithm to generate chemically reasonable 3D conformations because of its restrictive torsional and stereochemical constraints. Consequently, geometry optimization and subsequent PDBQT conversion could not be completed. The failed compounds were not corrected manually because this study evaluates the performance of the fully automated workflow. These observations are consistent with previously reported limitations of automated conformer-generation methods when applied to structurally constrained molecules.^15^^,^^16^^,^^17^

The observed success rate is likely attributable to the combination of standardization, protonation-state assignment, conformer generation, and force-field-based optimization incorporated into the workflow. Standardization removes inconsistencies from data sources. Protonation improves ligand representation under biological conditions. ETKDGv3^17^^,^^18^ conformer generation followed by MMFF94^19^ optimization produces stable 3D structures for docking. When MMFF94 parameters are missing, UFF^20^ optimization is applied. Together, these steps improve the probability that chemically reasonable structures can be generated from diverse compound collections. And Meeko^21^ successfully converted all the optimized structures into docking-ready PDBQT files.

Together, these results show that the sem_prep module is a reliable and scalable way to prepare diverse chemical datasets for virtual screening. By automating multiple preprocessing steps in one workflow, it reduces manual work, improves reproducibility, and removes a major barrier to large-scale docking studies.

### Validation of the extended workflow in molecular docking projects

The jamqvina module in jamdock-suite^14^ uses QuickVina 2^22^ (QVina 2) for molecular docking. Previous benchmarking studies reported that QVina 2 is approximately 2.3-fold faster than Vina on average, whereas Vina generally provides higher docking accuracy.^22^ Therefore, we selected Vina as the docking engine in sem_dock module to prioritize prediction quality. To evaluate the extended workflow, the prepared ligand library was docked against four protein targets obtained from the RCSB Protein Data Bank.^23^ The structures are PDB:1IE9,^24^ 3VHU,^25^ 4R38,^26^ and 1SQN.^27^ Receptor structures were prepared using the jamreceptor module from the jamdock-suite workflow.

For all protein targets, docking simulations were performed with parameters: an exhaustiveness of 32, a maximum number of binding modes of 9, and an energy range of 0–5 kcal/mol, indicating that all binding poses within 5 kcal/mol threshold of the best-scoring pose were preserved. All docking simulations were performed on a KVM-based virtual machine running Linux, equipped with an AMD EPYC 9334 32-core processor and 32 GB RAM, among which 30 CPU cores were utilized for parallel simulation.

In the original workflow, the jamqvina module uses a multithreading strategy, in which all the allocated CPUs perform a single docking job. In contrast, the sem_dock module implements ligand-wise CPU parallelization, in which the available processor cores are allocated with individual ligands and docked independently. This approach improves workload balance because every docking run is treated as its own task. It reduces idle CPU time caused by differences in ligand complexity and docking duration.

**Figure 2:**
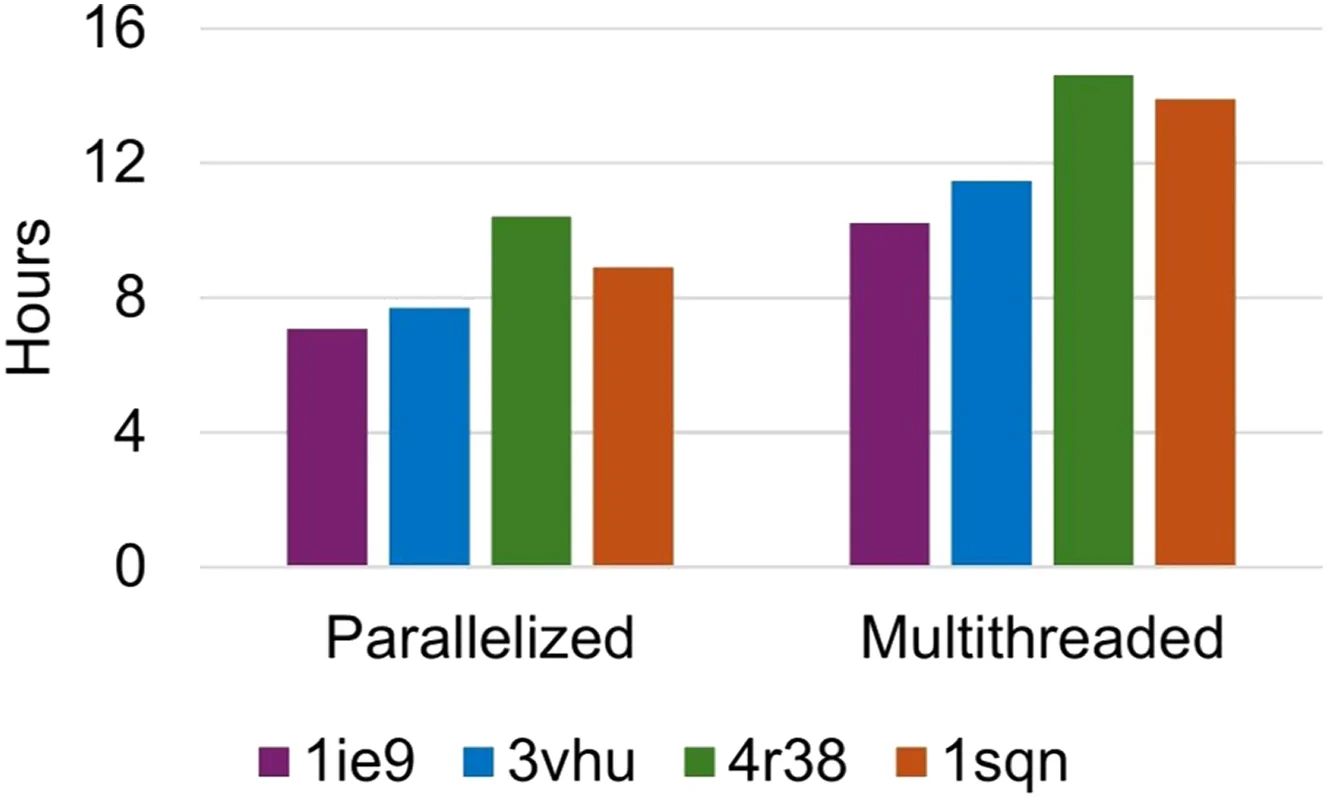
Comparison of docking runtimes using ligand-wise CPU parallelization and conventional multithreaded execution A library of 3,171 FDA-approved compounds was docked against four protein targets using identical docking parameters and hardware resources. Ligand-wise CPU parallelization implemented in the sem_dock module consistently reduced docking runtime compared with conventional multithreaded execution in AutoDock Vina.

As shown in Figure 2, using this strategy, docking of the prepared compound library took about 7–10 hours depending on the target. Under similar conditions, using AutoDock Vina’s conventional multithreaded execution with 30 CPU threads, docking of the same library required 10–15 hours. Overall, ligand-wise parallelization reduced docking time by approximately 30% while maintaining the identical docking parameters. Although the magnitude of improvement will depend on hardware specifications, docking parameters, and library composition, these results show that ligand-wise parallelization improves CPU use in large-scale virtual screening.

In addition to docking simulations, the workflow provides the sem_filter module, which automates ranking and filtering of compounds based on predicted binding affinity. The resulting datasets retain molecular descriptors and chemical representations. As a result, filtered datasets can be integrated into downstream cheminformatics analyses, machine-learning applications, and iterative screening campaigns. It also adapts SimScore calculations from jamrank module of jamdock-suite,^14^ which provides an additional measure of docking-pose convergence.

**Figure 3:**
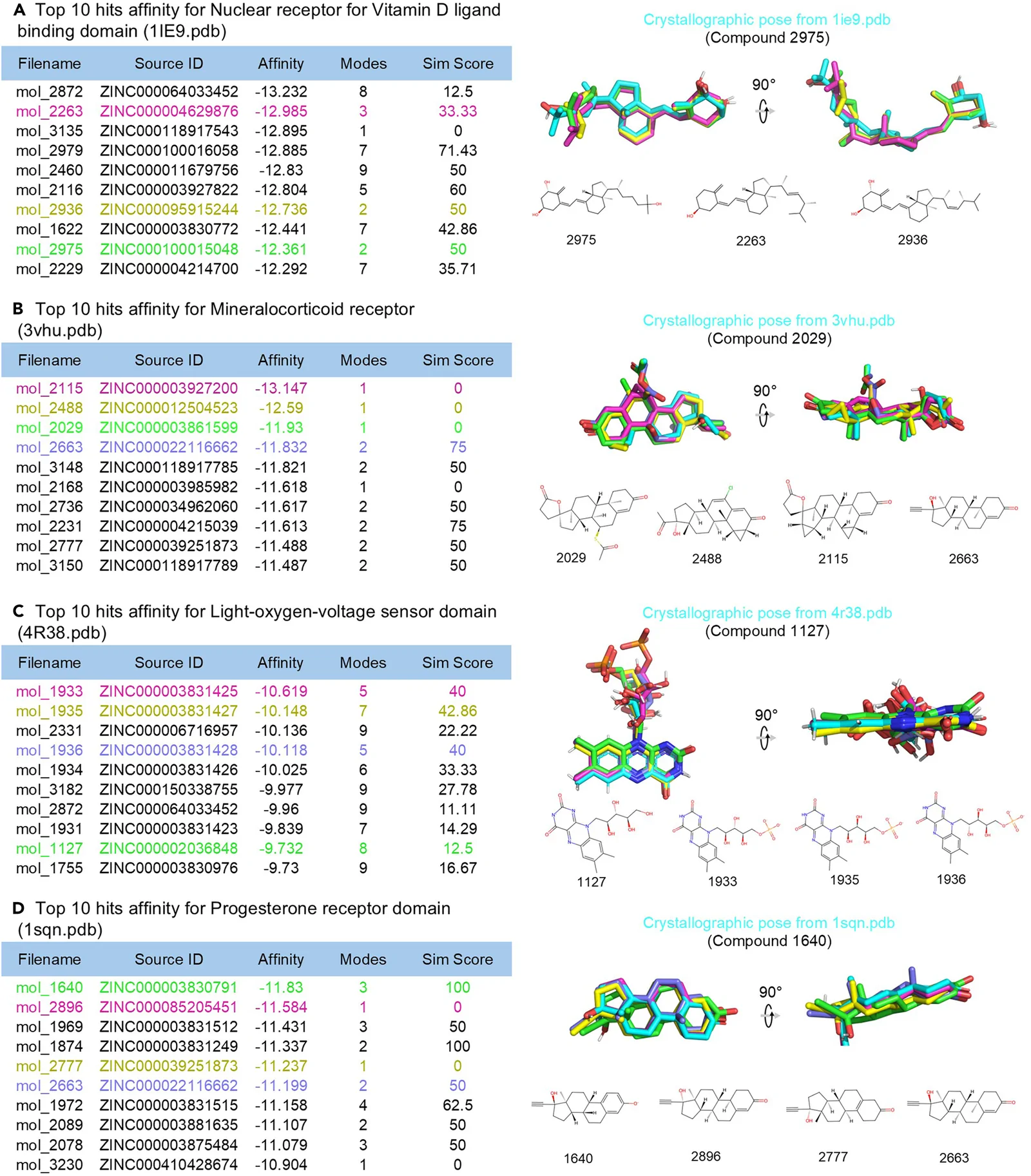
Docking performance across four protein targets showing binding affinity ranking and pose agreement with crystallographic ligands (A–D) Top-ranked compounds identified by docking against four protein targets: (A) Vitamin D nuclear receptor ligand-binding domain (PDB: 1IE9, chain A, pocket #5) (B) Mineralocorticoid receptor ligand-binding domain (PDB: 3VHU, chain A, pocket #1), (C) Light-oxygen-voltage (LOV) sensor domain (PDB: 4R38, chain A, pockets #2 and #3), and (D) Progesterone receptor ligand-binding domain (PDB: 1SQN, chain A, pocket #2). In each panel, the tables list the top ranked compounds with affinity (kcal/mol) scores including file name, source ID (ZINC), number of modes and SimScore. Some of the best poses of ligands compared to the crystallographic ligand are presented on the right side of the table with color-coded per compound.

To assess the practical utility of the workflow, top-ranked compounds identified by sem_filter module were examined for each protein target. In all four systems, the known co-crystallized ligands appeared among the top-ranked compounds. We further compared docking poses generated by sem_dock module with experimentally determined ligand conformations available in crystal structures. Visual comparison showed that the predicted poses are highly similar to experimentally observed binding modes (Figure 3). These results indicate that the changes made to ligand preparation and docking did not reduce the ability of the workflow to produce biologically meaningful results.

Overall, the extended workflow preserves the practical utility of the original jamdock-suite while expanding its use to user-defined compound collections.

## Discussion

### Applicability and limitations of the workflow

Although the semdock workflow enables large-scale screening from raw datasets, there are several limitations. sem_dock module uses rigid receptor docking and does not consider the flexibility of a protein. The accuracy of docking predictions may decrease for targets with highly dynamic binding sites. As a result, predicted binding affinities and poses should be considered approximations, best suited for early prioritization.

Also, the workflow, in its current state, considers a single protonation state as well as a single best conformer for each ligand. Although this improves computational efficiency and scalability, it reduces chemical sampling. As a result, some protonation states, tautomeric forms, stereochemical variants, or conformations relevant for binding may be excluded.

The workflow was designed to operate on standard CPU-based systems to maximize accessibility, but GPU acceleration is currently not implemented. In addition, since each simulation runs independently, separate memory is required for each process. Therefore, docking projects, especially blind docking with wide search spaces, may require substantial memory resources.

Finally, compound prioritization relies on docking scores, ranking, and optional SimScore calculations. These metrics help identify candidates but cannot separate true binders from false positives. Experimental validation remains essential, and compounds identified should be treated as hypotheses for further testing.

Despite these limitations, the extended workflow semdock removes a key limitation of the original jamdock-suite by allowing direct preparation of docking-ready ligands from mixed chemical datasets. Validation with FDA-approved compounds confirmed that ligand preparation is reliable. In addition, the ligand-wise CPU parallelization showed better performance in docking simulations. These improvements make the workflow more versatile, supporting screening projects that use public chemical libraries, curated collections, or user-defined libraries. At the same time, it preserves the automation and accessibility that made the original framework practical.

## Resource availability

### Lead contact

Requests for additional information and resources should be directed to and will be fulfilled by the lead contact, Jahangir Md. Alam (jahangir@btge.iu.ac.bd).

### Materials availability

This study presents a computational workflow and did not generate new physical materials.

### Data and code availability

The data used and generated during this study are available from the lead contact upon request. The source code is publicly available at GitHub (https://github.com/elrando/semdock) and Zenodo (https://doi.org/10.5281/zenodo.19998830).

## Acknowledgments

This work was supported by the Higher Education Acceleration and Transformation (HEAT) Project by the 10.13039/100015747University Grants Commission of Bangladesh, Bangladesh (PIN 13659; grant to J.M.A.). The authors would like to thank all co-investigators, colleagues, and lab members of the Molecular Biology Laboratory, Department of Biotechnology and Genetic Engineering, Islamic University for their kind support.

## Author contributions

S.E. conceptualized the workflow, developed the software, performed analyses, and wrote the original draft. J.M.A. supervised the study and reviewed and edited the manuscript. M.M.H., I.A., and M.A.H. contributed to data interpretation and manuscript review.

## Declaration of interests

The authors declare no competing interests.

## Declaration of generative AI and AI-assisted technologies in the writing process

During the preparation of this work, the authors used ChatGPT (OpenAI) to assist with language editing and manuscript formatting. After using this tool, the authors reviewed and edited the content as needed and take full responsibility for the content of the publication.

## STAR★Methods

### Key resources table

**Table:** 

REAGENT or RESOURCE	SOURCE	IDENTIFIER
**Deposited data**		

Bash and Python scripts for virtual screening pipeline	This paper; Zenodo: https://doi.org/10.5281/zenodo.19998830	GitHub: https://github.com/elrando/semdock.git
Compounds for virtual screening	ZINC Database	https://zinc.docking.org/
Crystal Structure of the vitamin D nuclear receptor ligand-binding domain	Tocchini-Valentini et al.^24^	PDB: 1IE9
Crystal structure of the mineralocorticoid receptor ligand-binding domain	Hasui et al.^25^	PDB: 3VHU
Crystal structure of the light-oxygen-voltage sensor domain	Rivera-Cancel et al.^26^	PDB: 4R38
Crystal structure of the progesterone receptor ligand-binding domain	Madauss et al.^27^	PDB: 1SQN

**Software and algorithms**		

Ubuntu 24.04 LTS	Ubuntu	https://ubuntu.com/blog/tag/ubuntu-2-04-lts
Python 3	Python	https://www.python.org/downloads
GNU Wget 1.21.4	GNU Project	https://www.gnu.org/software/wget
GNU nano 7.2	GNU Project	https://www.nano-editor.org/
curl 8.5.0	curl Project	https://curl.se/
Cmake 3.28.3	Kitware, Inc.	https://cmake.org/download/
Boost 1.83	Boost C++ Libraries	https://www.boost.org/
git 2.43.0	Git SCM	https://git.scm.com/
Conda 26.1.0	ANACONDA, Inc.	https://www.anaconda.com/docs/getting-started/miniconda/main
RDKit 2025.09.2	https://www.rdkit.org	https://doi.org/10.5281/zenodo.17495409
Meeko 0.7.1	Forli Lab	https://github.com/forlilab/Meeko
GNU Parallel	GNU Project; Zenodo: https://zenodo.org/records/19701238	https://www.gnu.org/software/parallel/
PyMOL 3.1.0 Open-Source	Schrödinger, Inc.	https://github.com/schrodinger/pymol-open-source
Dimorphite-DL	Durrant Lab	https://pypi.org/project/dimorphite-dl/
AutoDock Vina 1.2.7	Forli Lab (modified in this study)	Official: https://github.com/ccsb-scripps/AutoDock-Vina Modified: https://github.com/elrando/AutoDock-Vina

### Method details

#### Preparation of ligand library

For workflow validation, semdock workflow was installed in a Linux environment with all required software dependencies as described in Data S1. For ligand preparation, a library of 3230 FDA-approved compounds was downloaded from the ZINC database in CSV format. The exact CSV file used for workflow validation is provided in Data S7. The downloaded file contained compound identifiers and canonical SMILES representations, which served as input to the sem_prep module (Data S2). The module accepts input datasets in SDF, SMILES or CSV format. For CSV input, the only required field is a column containing SMILES representations. As a result, downloaded datasets from public resources such as ZINC, PubChem, or ChEMBL can generally be used without any preprocessing, since they already include a SMILES column.

The ligand preparation consisted of standardization, protonation-state assignment, three-dimensional conformer generation, geometry optimization, and conversion to docking-ready PDBQT format. These steps were integrated within the sem_prep module. The module utilizes three supporting scripts (Data S2): input_handler.py for parsing and standardizing input datasets, protonate.sh for protonation, and generate_3d.py for conformer generation and energy minimization. Molecular structures were first standardized using RDKit,^28^ followed by protonation-state assignment using Dimorphite-DL^29^ at pH 7.4. A single protonation state was generated for each compound. Conformers were generated using the ETKDGv3^18^ algorithm implemented in RDKit, and then energy-minimized using MMFF94,^19^ with UFF^20^ used as a fallback when MMFF94 parameters were unavailable. Finally, optimized structures were converted to PDBQT format using Meeko^21^ for docking simulations. Throughout the workflow, compound identifiers were preserved to ensure traceability during both docking and post-docking analysis, and processing logs were generated at every stage to record successful and failed preparation steps.

#### Molecular docking

Four protein structures were selected from the RCSB Protein Data Bank (PDB: 1IE9, 3VHU, 4R38, and 1SQN) for workflow validation. Receptor preparation was performed using the jamreceptor module (Data S3) from the jamdock-suite workflow described by Barbosa Pereira et al.^14^ Using the jamreceptor module, receptor structures were cleaned, assigned Gasteiger charges, and converted to PDBQT format. Also, binding pockets were identified using fpocket^30^ implemented in jamreceptor. Docking grid parameters were generated from the selected binding pockets and used for subsequent calculations.

Molecular docking was performed using the sem_dock module (Data S4) employing Vina as the docking engine. For all targets, docking simulations were performed using an exhaustiveness value of 32, a maximum of nine binding modes, and an energy range of 5 kcal/mol above the best-ranked pose. The module distributes ligands across available processor cores and performs docking calculations independently for each ligand. All docking calculations were performed on a KVM-based Linux virtual machine equipped with an AMD EPYC 9334 32-core processor and 32 GB RAM. Thirty CPU cores were allocated for docking simulations. To benchmark computational performance, docking calculations were performed using both ligand-wise parallel execution and AutoDock Vina’s conventional multithreaded execution under identical hardware conditions. Docking produced ligand poses in PDBQT format along with AutoDock Vina log files. Full execution procedures, workflow settings, and options are provided in Data S1.

#### Post-docking analysis

Post-docking analysis was performed using the sem_filter module (Data S5). The module processes AutoDock Vina output files and generates ranked compound lists based on the predicted binding affinity. Results can be analyzed either by applying a binding-affinity threshold or selecting a specific number of top-ranked compounds.

After the execution of the sem_filter module, three CSV datasets were generated. Each of the files consists of ligand file name, source database, source identifier (ZINC ID, PubChem CID, etc.), and binding affinity. One file contains the binding affinity for all docked ligands; another contains the filtered or ranked compounds only. The third file contains filtered compounds together with additional molecular descriptors including molecular weight (MW), topological polar surface area (TPSA), hydrogen bond donors (HBD), hydrogen bond acceptors (HBA), rotatable bonds (RotB), and water partition coefficient (LogP).

The module also implements an optional SimScore calculation adapted from the jamrank module of the jamdock-suite.^14^ SimScore measures how similar the docking poses are for the same ligand, giving an indication of pose convergence. It is used as an extra metric to help prioritize compounds. Detailed filtering procedures and execution options are provided in Data S1.

### Quantification and statistical analysis

Ligand preparation success rate was calculated as the percentage of compounds successfully converted into docking-ready PDBQT structures relative to the total number of compounds in the validation library. Docking performance was assessed by recording the total execution time for ligand-wise CPU parallelization and comparing it with AutoDock Vina’s standard multithreaded mode under identical hardware. Predicted docking poses were visually compared with ligand conformations from crystal structures. Structural overlays and figure preparation were carried out using PyMOL.

## Supplemental information

Data S1. Detailed implementation procedures for the semdock workflow, related to Preparation of ligand library

Data S2. Code for the sem_prep module and supporting scripts, related to Preparation of ligand library

Data S3. Code for the jamreceptor script, related to Post-Docking analysis

Data S4. Code for the sem_dock script, related to Molecular Docking

Data S5. Code for the sem_filter script, related to Molecular Docking

Data S6. Code for the fail_check script, related to Molecular Docking

Data S7. CSV file of the FDA-approved compound library used for workflow validation, related to Preparation of ligand library
