# Supplementary material for: An extension of jamdock-suite for virtual screening from raw chemical datasets
Source: STAR Protoc. 2026 Sep 7;7(3):104822. doi: 10.1016/j.xpro.2026.104822 (PMC13615501; doi:10.1016/j.xpro.2026.104822)
Supplement: Supplementary file 1 [file mmc1.pdf]

Data S1. Detailed implementation procedures for the semdock workflow

Overview of the workflow

The semdock workflow extends the jamdock-suite virtual screening pipeline by enabling direct processing of user-supplied chemical datasets and automated preparation of docking-ready ligands. The workflow consists of four modules that collectively perform ligand preparation, receptor preparation, molecular docking, and post-docking analysis.

Table S1. Overview of the semdock workflow.

Module	Purpose	Input	Output
sem_prep	Preparation of docking-ready ligands	SDF, SMILES or CSV files containing chemical structures	Standardized structures, protonated structures, optimized 3D conformers, and PDBQT files
jamreceptor	Receptor preparation and binding site identification	Protein structure in PDB format	Receptor PDBQT file, docking configuration file, and grid box definition
sem_dock	Molecular docking using AutoDock Vina	Prepared ligand library and receptor files	Docked ligand poses and docking log files
sem_filter	Post-docking analysis and compound prioritization	Docking output files and prepared ligand library	Ranked compound lists, filtered datasets, molecular descriptors, and optional SimScore calculations

System setup and installing software dependencies

This protocol is designed for use on Linux-based system using a Conda environment for simplified dependency management. We have also tested the workflow on Windows 11 using Windows Subsystem for Linux (WSL), where it executed without modification. Installation guidance for Windows (WSL) is available in Barbosa et al. [S1]. Although the workflow is expected to be compatible with other Unix-like operating systems such as macOS, this was not validated in the present study.

1. Update the system and install essential packages:

```
$ sudo apt update && sudo apt upgrade -y
$ sudo apt install -y build-essential nano wget curl git cmake
libboost-all-dev
```

   You will be required to input you superuser password.

2. Install Conda and create an environment
   a. Download the Miniconda and run the installer and complete the installation:

```
$ wget https://repo.anaconda.com/miniconda/Miniconda3-latest-Linux-
x86_64.sh
$ bash ./Miniconda3-latest-Linux-x86_64.sh
$ source ~/.bashrc
```

   Note: During installation, the user is prompted to accept the license agreement and confirm installation settings (e.g., installation directory and shell initialization). Accept the license and

allow Conda to modify the shell environment when prompted (e.g., select "Yes" for conda init). This ensures that the conda command is available in the terminal after installation.

b. Create an environment:

```
$ conda create --name semdock python=3.10
$ conda activate semdock
$ conda config --env --add channels conda-forge
```

3. Installing the software dependencies

a. Install RDKit, Meeko, GNU parallel and PyMOL using conda-forge:

```
$ conda install -c conda-forge rdkit meeko parallel pymol-open-
source
```

Note: For HPC clusters or command-line-only virtual machines, it is recommended to exclude PyMOL from installation, as these environments typically lack graphical support. Installing pymol-open-source may fail or stall due to missing visualization dependencies.

Note: You will encounter a GNU Parallel citation reminder when executing scripts that use parallel, to suppress this reminder, run the following command once:

```
$ parallel --citation
```

b. Install Dimorphite-DL

```
$ pip install dimorphite_dl
```

4. Install AutoDockTools

a. Create a directory for installation

```
$ mkdir ~/programs
$ cd ~/programs
```

b. Download and extract MGLTools:

```
$ wget
https://ccsb.scripps.edu/mgltools/download/491/mgltools_Linux-
x86_64_1.5.7.tar.gz
$ tar -zxf mgltools_Linux-x86_64_1.5.7.tar.gz
```

c. Install AutoDockTools:

```
$ cd mgltools_x86_64Linux2_1.5.7
$ ./install.sh
```

d. Configure shell environment for AutoDockTools

```
$ echo 'export
PATH=$HOME/programs/mgltools_x86_64Linux2_1.5.7/bin:$PATH' >>
~/.bashrc
$ source ~/.bashrc
```

5. Install fpocket

a. Clone fpocket repository, build and install

```
$ cd ~/programs
$ git clone https://github.com/discngine/fpocket.git
$ cd fpocket
$ make
$ sudo make install
```

6. Install AutoDock Vina
   a. Clone the AutoDock Vina repository and build:

```
$ cd ~/programs
$ git clone https://github.com/elrando/AutoDock-Vina
$ cd AutoDock-Vina/build/linux/release
$ make
```

Note: The official AutoDock Vina repository (https://github.com/ccsb-scripps/AutoDock-Vina) requires compilation with C++11, which may cause compatibility issues on some systems. To ensure stable compilation, we used a modified fork in which the compiler standard was updated to C++14. This modification does not affect docking functionality but improves build compatibility across environments.

   b. Configure shell environment for Vina:

```
$ echo "export PATH=$HOME/programs/AutoDock-
Vina/build/linux/release:\$PATH" >> ~/.bashrc
$ source ~/.bashrc
```

7. Download the protocol scripts and configure the environment
   a. Clone the semdock repository:

```
$ cd ~/programs
$ git clone https://github.com/elrando/semdock.git
$ cd semdock
```

   b. Make the scripts executable:

```
$ chmod +x sem_* jamreceptor fail_check
$ chmod +x py/*.py
$ chmod +x lib/*.sh
```

   c. Add semdock to shell's path:

```
$ echo "export PATH=$HOME/programs/semdock:\$PATH" >> ~/.bashrc
$ source ~/.bashrc
```

Note: This enables sem_prep, jamreceptor, sem_dock and sem_filter to be called from any terminal window.

Step-by-step execution details

This section presents detailed instructions for using the protocol. The protocol begins with an input compound library in SDF, SMILES, or CSV format. The procedure to results consists of four steps: (1)

prepare compounds for docking; (2) prepare the receptor and define a grid box; (3) perform docking to the prepared receptor; (4) extract the results and filter compounds. For demonstration, we applied the workflow to four study systems using an FDA-approved compound library.

Preparing Ligands

Use the sem_prep (Data S2) script to convert input ligands into docking-ready PDBQT files compatible with Vina, QuickVina, and Vina-GPU. This script performs standardization, protonation, 3D conformer generation, geometry optimization, and conversion to docking ready PDBQT files.

1. Activate the conda environment
   a. Open a terminal window and activate semdock environment

```
$ conda activate semdock
```

2. Create a working directory:
   a. Create a new directory for your project and navigate into the directory

```
$ mkdir ~/virtual_screening_project
$ cd ~/virtual_screening_project
```

   Note: Replace "virtual_screening_project" with a preferred project name.

3. Prepare the ligand input file
   a. Place the ligand input file (SDF, SMILES, or CSV format) in the working directory
   b. Make sure that the file contains one or more compounds and follows the correct formatting rules:
      i. SDF: multi-molecule structure file with 2D or 3D molecular structures
      ii. SMILES (.smi): For each compound, one SMILES string per line
      iii. CSV: tabular file containing at least one column with valid SMILES strings

Note: For CSV files, the SMILES column is essential as this is the information with which the protocol begins ligand preparation. The script automatically recognizes commonly used column names such as "smiles", "SMILES", "canonical_smiles", "smile", and "SMILE". Missing or malformed SMILES entries can cause failures during ligand preparation.

4. Launch the ligand preparation script sem_prep:
   a. In the terminal, run the script in the working directory by providing the required inputs:
      i. Input file (`--input`)
      ii. pH value for protonation (`--ph`)
      iii. Number of CPUs to use (`--cpu`) or fraction of available CPUs (`--cpu_fraction`)
      iv. Name of the library (`--out_dir`)

```
$ sem_prep --input fda.csv --ph 7.4 --cpu 30 --out_dir fda_lib
```

   Note: This command starts the ligand preparation workflow (Figure S1). Replace "fda.csv" with your input file name, and "fda_lib" with your preferred library name. Besides, we recommend choosing a CPU value lower than the total number of available CPU cores. This helps avoiding

excessive load during parallel execution. Otherwise, user can define CPU usage proportionally using the --cpu_fraction option. For example:

```
$ sem_prep --input fda.csv --ph 7.4 --cpu_fraction 0.8 --out_dir
fda_lib
```

This uses approximately 80% of the total available CPU cores and keeps the system responsive during long processing simulations.

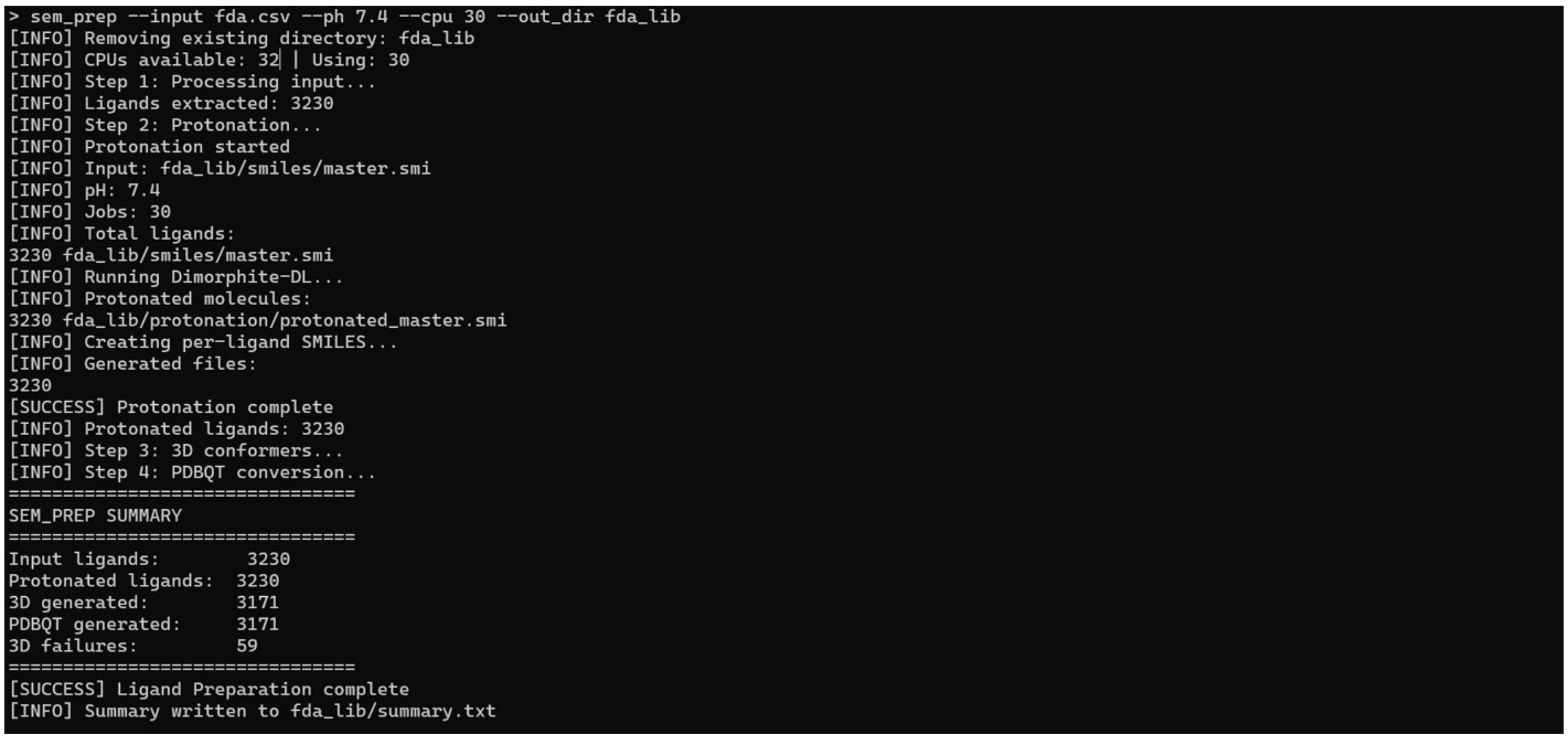

```
> sem_prep --input fda.csv --ph 7.4 --cpu 30 --out_dir fda_lib
[INFO] Removing existing directory: fda_lib
[INFO] CPUs available: 32 | Using: 30
[INFO] Step 1: Processing input...
[INFO] Ligands extracted: 3230
[INFO] Step 2: Protonation...
[INFO] Protonation started
[INFO] Input: fda_lib/smiles/master.smi
[INFO] pH: 7.4
[INFO] Jobs: 30
[INFO] Total ligands:
3230 fda_lib/smiles/master.smi
[INFO] Running Dimorphite-DL...
[INFO] Protonated molecules:
3230 fda_lib/protonation/protonated_master.smi
[INFO] Creating per-ligand SMILES...
[INFO] Generated files:
3230
[SUCCESS] Protonation complete
[INFO] Protonated ligands: 3230
[INFO] Step 3: 3D conformers...
[INFO] Step 4: PDBQT conversion...
================================
SEM_PREP SUMMARY
================================
Input ligands:         3230
Protonated ligands:   3230
3D generated:         3171
PDBQT generated:      3171
3D failures:          59
================================
[SUCCESS] Ligand Preparation complete
[INFO] Summary written to fda_lib/summary.txt
```

Figure S1. Execution of sem_prep for automated ligand preparation; The workflow performs compound standardization, protonation at user-defined pH, 3D conformer generation, geometry optimization, and conversion to docking-ready PDBQT format.

5. Allow the script to complete ligand preparation:
   a. Here, sem_prep automatically performs ligand standardization, protonation-state assignment, conformer generation, geometry optimization, and PDBQT conversion.

   Note: The time to complete the process depends on the number of compounds in the input file and available computational resources. Large compound libraries may require several hours to complete.

   b. Verify the generated output files in the directory specified in "`--out_dir`". Expected output directories include: smiles, protonation, 3D, pdbqt and logs. Each of the folders contains the outputs of its respective processing stage. Log files are generated through the workflow to track failures.
   c. Review the generated id_map.csv file. This file records the relationship between original compound identifiers (e.g., ZINC ID, PubChem CID, or custom names) and internally generated file names, which enables traceability during docking and post-docking analysis.

   Note: The id_map.csv file should be retained for downstream analysis, as it is required to map results back to the original compounds.

Preparing Receptor

In this protocol, receptor preparation is performed using the jamreceptor script (see Data S3), adapted from the jamdock-suite workflow described by Barbosa Pereira et al. [S1].

6. Create a receptor-specific working directory
   a. Create a new directory for the target receptor

```
$ mkdir ~/virtual_screening_project/1sqn
$ cd ~/virtual_screening_project/1sqn
```

Note: Replace “1sqn” with your target receptor name.

7. Prepare your input file.
   a. Place your receptor structure file in PDB format (for example: 1SQN.pdb) inside the working directory and un jamreceptorExecute the receptor preparation script:

```
$ jamreceptor
```

When prompted, input the name of the PDB file you want to process (e.g., 1SQN.pdb)

   b. Choose one or more chains of your interest to retain from input PDB file (e.g. for 1SQN select chain A, pocket 2)

Note: At this step, the script removes non-selected chains and non-protein atoms, adds Gasteiger charges, and converts the cleaned receptor structure into docking-ready PDBQT format. Processing time depends on receptor size and system performance.

8. Analyze binding pockets with fpocket
   a. The script will identify ligand-binding pockets
   b. Select the desired pocket(s)
   c. Define grid box padding

Note: Detailed instructions for receptor preparation and pocket selection are described in Barbosa Pereira et al. [S1]. This protocol follows the same receptor preparation workflow with minor output standardization for compatibility with sem_dock.

9. Verify the generated output files
   a. After receptor preparation, confirm that the following files are present:
      i. receptor.pdbqt (docking-ready PDBQT)
      ii. conf.txt (grid box dimensions for docking)
      iii. grid_box.py (PyMOL script to visualize grid box)

Note: The grid box information file (conf.txt) and receptor PDBQT file (receptor.pdbqt) must remain in the same working directory where docking will be performed.

Optional: An experienced user may use their own pre-prepared receptor file (receptor.pdbqt) and configuration file (conf.txt) by placing both files in the working directory.

Performing Docking

To begin docking the prepared compounds, use the sem_dock script (Data S4). This will automate molecular docking using the receptor structure (receptor.pdbqt) and grid box parameters provided in configuration file (conf.txt). sem_dock supports CPU-parallel execution, and interrupted runs can be resumed automatically when the same output directory is reused.

To keep docking outputs organized by receptor, we recommend running the script inside the receptor-specific working directory. And the required config and PDBQT file exist there by default due to the previous workflow.

10. Launch the docking script:
    a. Open a terminal window and navigate to the receptor directory

```
$ cd ~/virtual_screening_project/1sqn
```

    b. Execute the process by providing required inputs:
        i. Configuration file (--config)
        ii. Directory of prepared ligand used in the sem_prep (--lig_dir)
        iii. Number of CPUs to use (--cpu) or fraction of available CPUs (--cpu_fraction)
        iv. Name of the directory (--out_dir)

```
$ sem_dock --config conf.txt --lig_dir
~/virtual_screening_project/fda_lib --cpu 30 --out_dir
~/virtual_screening_project/1sqn
```

Note: Ensure that the configuration file (conf.txt), and receptor PDBQT file (receptor.pdbqt) exist in the working directory.

Note: This command starts the docking process with the prepared ligands located in `~/virtual_screening_project/fda_lib` directory. To avoid confusion, we used full location for the ligand directory (`--lig_dir`) and output directory (`--out_dir`). However, users may use relative directory notations. As the script is being executed at the receptor directory (`~/virtual_screening_project/1sqn`) user can use:

```
$ sem_dock --config conf.txt --lig_dir ../fda_lib --cpu 30 --out_dir
.
```

Note: By default, these commands perform docking using AutoDock Vina default parameters:
i. Exhaustiveness = 8
ii. Maximum number of poses = 9
iii. Energy range = 3

Users may optionally change these parameters by using command line arguments, for example:

```
$ sem_dock --config conf.txt --lig_dir
~/virtual_screening_project/fda_lib --exhaustiveness 32 --max_poses
20 --energy_range 5 --cpu 30 --out_dir
~/virtual_screening_project/1sqn
```

Also, the `--cpu_fraction` is usable as defined at step 3.

11. Monitor docking progress (Figure S2):
    a. Let the script run automatically
    b. Observe live updates printed to the terminal, including:
        i. Number of CPU cores in use
        ii. Total number of ligands
        iii. Number of compounds docked
        iv. Estimated time remaining
        v. Docking rate per minute

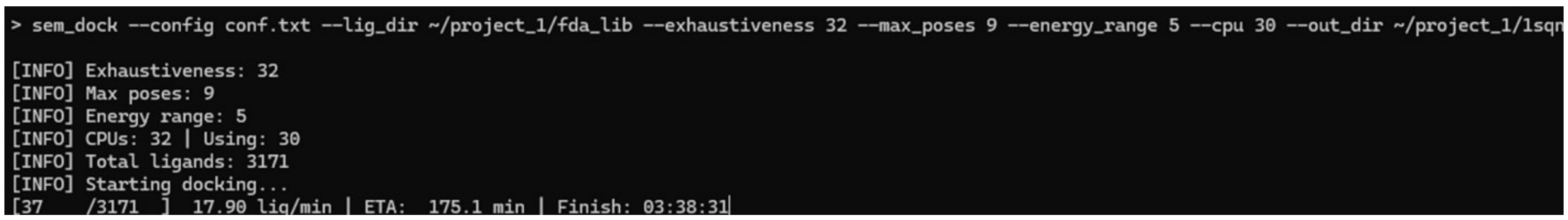

Figure S2. CPU-parallelized docking execution with sem_dock script and real-time progress monitoring; Terminal output from sem_dock showing parallel docking execution. Each ligand is processed independently on a single CPU core, enabling efficient resource utilization. Real-time updates include number of processed ligands, docking rate, CPU usage, and estimated completion time.

12. Resume interrupted docking runs

If docking is interrupted, rerun the same command in the same working directory. The script will automatically detect completed docking jobs and skip them.

13. Verify docking output files:
    a. Confirm that the output directory contains:
        i. vina_out/ –docked ligand structures
        ii. vina_logs/ – AutoDock Vina log files
        iii. ligands.txt – list of processed ligands
        iv. failed_docking.txt – records failed docking attempts, if any

    Note: This protocol uses CPU-based parallelization where each ligand is docked independently on a single CPU core. For example, using --cpu 30 launches 30 parallel docking jobs. Since each docking job requires memory, sufficient random-access memory (RAM) is necessary for stable execution. In our example, using a grid size of 22 Å × 23 Å × 26 Å required about 12.4 GB of RAM for 30 parallel docking jobs. For blind docking to work seamlessly using a large grid box size (>100 Å on per side), we recommend a system to have approximately 2× N (number of CPUs) GBs of RAM.

Filtering Docking Results

To analyze the docked compounds based on their binding affinities, use the sem_filter script (Data S5). This script extracts binding affinity from Vina output files and filters compounds using a threshold-based or top-hit selection method.

14. Execute the sem_filter script:
    a. Open a terminal window in the receptor directory and run the command with inputs:
        i. Docking directory (`--dock_dir`)
        ii. Ligand directory (`--prep_dir`)

iii. Binding affinity threshold to filter (`--threshold`) or top-hit selection (`--top`)

Example using threshold-based filtering using binding affinity score (≤ -10.0 kcal/mol):

```
$ cd ~/virtual_screening_project/1sqn
$ sem_filter --dock_dir ~/virtual_screening_project/1sqn --prep_dir
~/virtual_screening_project/fda_lib --threshold -10.0
```

15. Alternative filtering strategies
    a. Use top-hit ranking instead of threshold filtering:

```
$ sem_filter --dock_dir ~/virtual_screening_project/1sqn --prep_dir
~/virtual_screening_project/fda_lib --top 100
```

This command selects the top 100 compounds with strongest predicted binding affinity.

b. Enable SimScore calculation by adding `--simscore` flag:

```
$ sem_filter --dock_dir ~/virtual_screening_project/1sqn --prep_dir
~/virtual_screening_project/fda_lib --threshold -10.0 --simscore
```

Note: The script, when --simscore is enabled, evaluates convergence and diversity of predicted docking poses by analyzing RMSD variation. It is calculated as:

$$SimScore = \frac{1}{2} \times \left(P_{RMSD\ lower\ bound<1.6\ Å} + P_{RMSD\ upper\ bound<3.2\ Å}\right)$$

where, $P_{RMSD\ lower\ bound<1.6Å}$ is the percentage of modes with a root mean square deviation (RMSD) lower bound < 1.6 Å relative to the best-scoring (lowest-energy) pose. $P_{RMSD\ upper\ bound<3.2Å}$ is the percentage of docking modes with RMSD upper <3.2 Å relative to the best scoring pose.

GNU nano 7.2 Filtered_Results

Ligand	Source	SourceID	Affinity	TotalModes	SimScore
mol_1640	ZINC	ZINC000003830791	-11.83	3	67
mol_2896	ZINC	ZINC000085205451	-11.584	1	0
mol_1969	ZINC	ZINC000003831512	-11.431	3	17
mol_1874	ZINC	ZINC000003831249	-11.337	2	50
mol_2777	ZINC	ZINC000039251873	-11.237	1	0
mol_2663	ZINC	ZINC000022116662	-11.199	2	0
mol_1972	ZINC	ZINC000003831515	-11.158	4	38
mol_2089	ZINC	ZINC000003881635	-11.107	2	0
mol_2078	ZINC	ZINC000003875484	-11.079	3	17
mol_3230	ZINC	ZINC000410428674	-10.904	1	0
mol_1642	ZINC	ZINC000003830793	-10.893	2	50
mol_1300	ZINC	ZINC000003814395	-10.87	1	0
mol_2191	ZINC	ZINC000004097304	-10.848	2	0
mol_2290	ZINC	ZINC000005167143	-10.825	1	0
mol_1273	ZINC	ZINC000003812897	-10.821	3	33
mol_2115	ZINC	ZINC000003927200	-10.811	1	0
mol_2251	ZINC	ZINC000004428529	-10.781	2	25
mol_1613	ZINC	ZINC000003830763	-10.775	6	42
mol_1302	ZINC	ZINC000003814422	-10.733	2	25
mol_2316	ZINC	ZINC000006067483	-10.702	2	25
mol_2091	ZINC	ZINC000003881958	-10.65	1	0
mol_2127	ZINC	ZINC000003938695	-10.626	1	0
mol_1641	ZINC	ZINC000003830792	-10.618	4	38
mol_2488	ZINC	ZINC000012504523	-10.589	1	0

Figure S3. Post-docking filtering and ranking of candidate compounds; Example output of sem_filter showing filtered compounds ranked by binding affinity. The results consist of ligand identifiers (ligand file name, source and source ID), binding scores, number of docking modes, and optional SimScore values.

16. Verify output files

a. By default, the script generates an analysis/ directory inside the docking output directory (`--dock_dir`).
b. Confirm that the following output files are generated:
   i. Results.csv
   ii. Filtered_Results.csv
   iii. Filtered_Features.csv
c. Visualize the filtered results (Figure S3):

```
$ nano Filtered_Results.csv
```

Note: The Results.csv contains the docking score for all successfully docked compounds. Filtered results, either by threshold or top ranking, are stored in Filtered_Results.csv (Figure S3)

Troubleshooting

Problem 1

sem_prep may fail to recognize compounds in CSV input files.

Potential solution

This can occur due to not having a valid SMILES column in the CSV file, or the column header is not recognized by the script. Make sure that the CSV file contains at least one column with valid SMILES strings. Common supported column names include SMILES, smiles, smile, canonical_smiles, and similar variants. Remove empty rows and verify that each entry contains a chemically valid SMILES string.

Problem 2

The generated id_map.csv file does not correctly capture ligand source and identifier information from custom CSV input files.

Potential solution

This occurs when multiple identifier-related columns (e.g., Ligand, Batch, CID) are present in the CSV file, and the pipeline cannot capture the ligand source and original identifier. As a result, ligands are labeled as “Custom” with missing or incorrect Source and SourceID fields. This may occur even when the CSV contains valid identifiers, because the pipeline does not recognize non-standard column names. Make sure that the identifier column is clearly distinguishable and preferably named using standard conventions such as: PubChem_CID, ZINC_ID, ChEMBL_ID. Avoid using generic column names such as ID, Name, or Ligands as these are not recognizable by the pipeline.

Problem 3

Some ligands fail during 3D conformer generation

Potential solution

Compounds with unusual valence states, highly strained ring systems, organometallic structures, unstable charged groups, or unsupported chemistries may fail during ETKDGv3 conformer generation, MMFF94 minimization, or UFF fallback.
To identify failed compounds, run the following command to use the fail_check script (Data S6):

```
$ fail_check --prep_dir <path_to_sem_prep_output_directory>
```

If necessary, manually prepare problematic ligands using external cheminformatics tools or remove them from screening library if they are not essential for the study. A small percentage (0-5%) of failures is expected for chemically diverse large-scale libraries.

Problem 4

sem_dock may stop unexpectedly, or the system may become unresponsive during docking.

Potential solution

This occurs when the number of parallel docking jobs exceeds available system memory (RAM), especially for large grid boxes. Reduce the number of CPU cores used by lowering the --cpu value or using --cpu_fraction to limit resource consumption.

For blind docking with very large grid boxes (>100 Å per side), ensure that sufficient RAM is available. As a practical guideline, we recommend approximately:

$RAM \approx 2 \times N$ GB
where N is the number of CPU cores used for parallel docking.

If available RAM is highly limited, users may also modify the cpu value inside conf.txt together with the --cpu value during command execution. For example, for blind docking simulation with box size larger than 100 Å per side on system with 8 GB RAM, with 8 core CPU, user may set

`cpu = 2`	inside conf.txt
`--cpu 4`	during docking execution

This runs 4 parallel ligands docking jobs, with each using 2 CPU cores. The multiplication of these values (cpu × --cpu) must not exceed the total available CPU cores in the system. This strategy improves stability while making better use of available computational resources.

Problem 5

Interrupted docking does not resume correctly.

Potential solution

This may be caused by using a different output directory or a modified ligand directory, which prevents sem_dock from detecting previously completed docking files. Rerun the docking command using the same --out_dir directory and the same ligand preparation directory (--lig_dir). The script resumes by checking existing docking output files inside the output directory. Changing the output directory will start a new docking run rather than resuming the previous one.

References

[S1] Barbosa Pereira, P.J., Ripoll-Rozada, J., Macedo-Ribeiro, S., and Manso, J.A. (2025). Protocol for an automated virtual screening pipeline including library generation and docking evaluation. STAR Protocols *6*, 104161. https://doi.org/10.1016/j.xpro.2025.104161.
