# Supplementary material for: An extension of jamdock-suite for virtual screening from raw chemical datasets
Source: STAR Protoc. 2026 Sep 7;7(3):104822. doi: 10.1016/j.xpro.2026.104822 (PMC13615501; doi:10.1016/j.xpro.2026.104822)
Supplement: Supplementary file 2 [file mmc2.pdf]

# Data S2. Code for the sem_prep and supporting scripts

The sem_prep script depends on three additional scripts (input_handler.py, protonate.sh, generate_3d.py).

**sem_prep:**

```
#!/usr/bin/env bash

set -euo pipefail

# ============================================================
# SEM_PREP — Ligand Preparation Pipeline (Improved)
# ============================================================

# ----------------------------
# Environment safety (IMPORTANT FIX)
# ----------------------------
export OMP_NUM_THREADS=1
export MKL_NUM_THREADS=1
export OPENBLAS_NUM_THREADS=1

# ----------------------------
# Setup
# ----------------------------
SCRIPT_DIR="$(cd "$(dirname "${BASH_SOURCE[0]}")" && pwd)"

PH=7.4
CPU=""
CPU_FRACTION=0.8
OUTDIR="prep_out"
RESUME=false

# ----------------------------
# Usage
# ----------------------------
usage() {
cat << EOF

============================================================
 SEM_PREP — Ligand Preparation Pipeline
============================================================
```

```
USAGE:
  sem_prep --input FILE --out_dir DIR [options]

REQUIRED:
  --input FILE
  --out_dir DIRECTORY

OPTIONAL:
  --ph FLOAT            (default: 7.4)
  --cpu INT
  --cpu_fraction FLOAT   (default: 0.8)
  --resume              Resume existing run (no overwrite)

EOF
exit 1
}

# ----------------------------
# Argument parsing
# ----------------------------
while [[ $# -gt 0 ]]; do
    case $1 in
        -h|--help) usage ;;
        --input) INPUT="$2"; shift 2 ;;
        --ph) PH="$2"; shift 2 ;;
        --cpu) CPU="$2"; shift 2 ;;
        --cpu_fraction) CPU_FRACTION="$2"; shift 2 ;;
        --out_dir) OUTDIR="$2"; shift 2 ;;
        --resume) RESUME=true; shift ;;
        *) usage ;;
    esac
done

[[ -z "${INPUT:-}" ]] && usage

# ----------------------------
# Validate input
# ----------------------------
if [[ ! -f "$INPUT" ]]; then
    echo "[ERROR] Input file not found: $INPUT"
    exit 1
fi

if [[ ! -s "$INPUT" ]]; then
    echo "[ERROR] Input file is empty: $INPUT"
```

```
    exit 1
fi

# ----------------------------
# Handle output directory
# ----------------------------
if [[ "$RESUME" = false && -d "$OUTDIR" ]]; then
    echo "[INFO] Removing existing directory: $OUTDIR"
    rm -rf "$OUTDIR"
fi

# ----------------------------
# Directory structure
# ----------------------------
SMI_DIR="$OUTDIR/smiles"
PROT_DIR="$OUTDIR/protonation"
CONF_DIR="$OUTDIR/3D"
PDBQT_DIR="$OUTDIR/pdbqt"
LOG_DIR="$OUTDIR/logs"

mkdir -p "$SMI_DIR" "$PROT_DIR" "$CONF_DIR" "$PDBQT_DIR" "$LOG_DIR"

# ----------------------------
# CPU handling
# ----------------------------
TOTAL_CPU=$(nproc)

if [[ -n "${CPU:-}" ]]; then
    JOBS="$CPU"
else
    JOBS=$(python3 - <<EOF
import math
print(max(1, int($TOTAL_CPU * $CPU_FRACTION)))
EOF
)
fi

echo "[INFO] CPUs available: $TOTAL_CPU | Using: $JOBS"

# ============================================================
# STEP 1: INPUT PROCESSING
# ============================================================

echo "[INFO] Step 1: Processing input..."

python3 "$SCRIPT_DIR/py/input_handler.py" \
```

```
    --input "$INPUT" \
    --out_smi "$SMI_DIR/master.smi" \
    --id_map "$OUTDIR/id_map.csv" \
    > "$LOG_DIR/input.log" 2>&1

if [[ ! -s "$SMI_DIR/master.smi" ]]; then
    echo "[ERROR] No molecules extracted"
    exit 1
fi

TOTAL_LIGANDS=$(wc -l < "$SMI_DIR/master.smi")
echo "[INFO] Ligands extracted: $TOTAL_LIGANDS"

# ============================================================
# STEP 2: PROTONATION
# ============================================================

echo "[INFO] Step 2: Protonation..."

bash "$SCRIPT_DIR/lib/protonate.sh" \
    "$SMI_DIR/master.smi" \
    "$PROT_DIR" \
    "$PH" \
    "$JOBS"

if [[ ! -s "$PROT_DIR/protonated_master.smi" ]]; then
    echo "[ERROR] Protonation failed"
    exit 1
fi

PROTONATED_COUNT=$(wc -l < "$PROT_DIR/protonated_master.smi")
echo "[INFO] Protonated ligands: $PROTONATED_COUNT"

# ============================================================
# STEP 3: 3D GENERATION (RESUME SAFE)
# ============================================================

echo "[INFO] Step 3: 3D conformers..."

export SCRIPT_DIR CONF_DIR LOG_DIR

find "$PROT_DIR/protonated_smi" -name "*.smi" | \
parallel -j "$JOBS" --env SCRIPT_DIR --env CONF_DIR --env LOG_DIR '
infile={}
base=$(basename "$infile" .smi)
```

```
outfile="$CONF_DIR/${base}_3D.sdf"
logfile="$LOG_DIR/3D_${base}.log"

if [[ -s "$outfile" ]]; then
    exit 0
fi

python3 "$SCRIPT_DIR/py/generate_3d.py" \
    --input "$infile" \
    --outdir "$CONF_DIR" \
    > "$logfile" 2>&1 || echo "$base" >> "$LOG_DIR/3D_failed.txt"
'

THREED_COUNT=$(ls "$CONF_DIR"/*_3D.sdf 2>/dev/null | wc -l || true)

# ============================================================
# STEP 4: PDBQT CONVERSION (RESUME SAFE)
# ============================================================

echo "[INFO] Step 4: PDBQT conversion..."

export PDBQT_DIR LOG_DIR

find "$CONF_DIR" -name "*_3D.sdf" | \
parallel -j "$JOBS" --env PDBQT_DIR --env LOG_DIR '
infile={}
base=$(basename "$infile" _3D.sdf)

outfile="$PDBQT_DIR/${base}.pdbqt"
logfile="$LOG_DIR/meeko_${base}.log"

if [[ -s "$outfile" ]]; then
    exit 0
fi

mk_prepare_ligand.py \
    -i "$infile" \
    -o "$outfile" \
    > "$logfile" 2>&1

if [[ ! -s "$outfile" ]]; then
    echo "$base" >> "$LOG_DIR/pdbqt_failed.txt"
fi
'

PDBQT_COUNT=$(ls "$PDBQT_DIR"/*.pdbqt 2>/dev/null | wc -l || true)
```

```
# ============================================================
# FINAL SUMMARY (NEW FEATURE)
# ============================================================

SUMMARY_FILE="$OUTDIR/summary.txt"

echo "================================" | tee "$SUMMARY_FILE"
echo "SEM_PREP SUMMARY" | tee -a "$SUMMARY_FILE"
echo "================================" | tee -a "$SUMMARY_FILE"
echo "Input ligands:        $TOTAL_LIGANDS" | tee -a "$SUMMARY_FILE"
echo "Protonated ligands:  $PROTONATED_COUNT" | tee -a "$SUMMARY_FILE"
echo "3D generated:        $THREED_COUNT" | tee -a "$SUMMARY_FILE"
echo "PDBQT generated:     $PDBQT_COUNT" | tee -a "$SUMMARY_FILE"

if [[ -f "$LOG_DIR/3D_failed.txt" ]]; then
    FAIL3D=$(wc -l < "$LOG_DIR/3D_failed.txt")
    echo "3D failures:         $FAIL3D" | tee -a "$SUMMARY_FILE"
fi

if [[ -f "$LOG_DIR/pdbqt_failed.txt" ]]; then
    FAILPDBQT=$(wc -l < "$LOG_DIR/pdbqt_failed.txt")
    echo "PDBQT failures:      $FAILPDBQT" | tee -a "$SUMMARY_FILE"
fi

echo "================================" | tee -a "$SUMMARY_FILE"

echo "[SUCCESS] Ligand Preparation complete"
echo "[INFO] Summary written to $SUMMARY_FILE"
```

**input_handler.py:**

```
#!/usr/bin/env python3

import argparse
import csv
import re
import sys
from rdkit import Chem
from rdkit.Chem.MolStandardize import rdMolStandardize

# ============================================================
# CLI
# ============================================================
```

```
parser = argparse.ArgumentParser(
    description="Universal molecule input handler (robust, HTVS-
ready)"
)
parser.add_argument("--input", required=True)
parser.add_argument("--out_smi", required=True)
parser.add_argument("--id_map", required=True)
args = parser.parse_args()

ext = args.input.split(".")[-1].lower()

count, fail = 0, 0

# ============================================================
# Helpers
# ============================================================

def detect_source(raw_id):
    if not raw_id:
        return ("Custom", "")

    raw_id = str(raw_id).strip()

    if re.match(r'^ZINC', raw_id, re.I):
        return ("ZINC", raw_id)

    if re.match(r'^CHEMBL', raw_id, re.I):
        return ("ChEMBL", raw_id)

    if raw_id.isdigit():
        return ("PubChem", raw_id)

    return ("Custom", raw_id)


def clean_mol(mol):
    """Standard cleanup pipeline"""
    mol = rdMolStandardize.Cleanup(mol)
    mol = rdMolStandardize.FragmentParent(mol)
    Chem.SanitizeMol(mol)
    return mol


def detect_columns(headers):
    """Improved CSV column detection"""
```

```
smiles_col = None
id_col = None

id_priority = [
    "compound_cid",
    "compound cid",
    "cid",
    "pubchem_cid",
    "pubchem cid",
    "pubchem id",
    "pubchem"
    "zinc_id",
    "zinc id",
    "zinc",
    "chembl_id",
    "chembl id",
    "chembl",
    "id"
]

smiles_priority = [
    "smiles",
    "canonical_smiles",
    "canonical smiles",
    "smi"
]

lower_map = {h.lower(): h for h in headers}


 # SMILES detection
for key in smiles_priority:
    if key in lower_map:
        smiles_col = lower_map[key]
        break

# ID detection
for key in id_priority:
    if key in lower_map:
        id_col = lower_map[key]
        break

# Fallback fuzzy smiles
if smiles_col is None:
    for h in headers:
        if "smile" in h.lower():
```

```
                smiles_col = h
                break

    # Fallback ID = first column
    if id_col is None:
        id_col = headers[0]

    return smiles_col, id_col


# ============================================================
# Processing
# ============================================================

try:
    with open(args.out_smi, "w") as out_smi, open(args.id_map, "w") as
map_file:

        map_file.write("Ligand,Source,SourceID\n")

        # ------------------------------------------------------------
        # SDF
        # ------------------------------------------------------------
        if ext == "sdf":

            suppl = Chem.SDMolSupplier(args.input, sanitize=True,
removeHs=False)

            for i, mol in enumerate(suppl):

                if mol is None:
                    fail += 1
                    continue

                try:
                    mol = clean_mol(mol)

                    smi = Chem.MolToSmiles(mol, isomericSmiles=True)

                    raw_id = mol.GetProp("_Name") if
mol.HasProp("_Name") else f"mol_{i+1}"
                    source, sid = detect_source(raw_id)

                    count += 1
                    name = f"mol_{count}"
```

```
                    out_smi.write(f"{smi} {name}\n")
                    map_file.write(f"{name},{source},{sid}\n")

                except Exception as e:
                    print(f"[ERROR SDF] {e}", file=sys.stderr)
                    fail += 1

        # ---------------------------------------------------------------
        # SMI
        # ---------------------------------------------------------------
        elif ext == "smi":

            with open(args.input) as f:
                for i, line in enumerate(f):

                    parts = line.strip().split()
                    if not parts:
                        continue

                    smi = parts[0]
                    raw_id = parts[1] if len(parts) > 1 else
f"mol_{i+1}"

                    try:
                        mol = Chem.MolFromSmiles(smi)

                        if mol is None:
                            fail += 1
                            continue

                        mol = clean_mol(mol)

                        smi = Chem.MolToSmiles(mol,
isomericSmiles=True)

                        source, sid = detect_source(raw_id)

                        count += 1
                        name = f"mol_{count}"

                        out_smi.write(f"{smi} {name}\n")
                        map_file.write(f"{name},{source},{sid}\n")

                    except Exception as e:
                        print(f"[ERROR SMI] {e}", file=sys.stderr)
                        fail += 1
```

```
        # ---------------------------------------------------------------
        # CSV
        # ---------------------------------------------------------------
        elif ext == "csv":

            with open(args.input) as f:
                sample = f.read(5000)

            try:
                dialect = csv.Sniffer().sniff(sample)
            except:
                dialect = csv.excel

            with open(args.input) as f:
                reader = csv.DictReader(f, dialect=dialect)

                headers = reader.fieldnames

                if not headers:
                    raise ValueError("CSV has no headers")

                smiles_col, id_col = detect_columns(headers)

                if smiles_col is None:
                    raise ValueError("No SMILES column detected")

                for i, row in enumerate(reader):

                    smi = str(row.get(smiles_col, "")).strip()
                    raw_id = row.get(id_col, f"mol_{i+1}")

                    if not smi:
                        continue

                    try:
                        mol = Chem.MolFromSmiles(smi)

                        if mol is None:
                            fail += 1
                            continue

                        mol = clean_mol(mol)

                        smi = Chem.MolToSmiles(mol,
isomericSmiles=True)
```

```
                        source, sid = detect_source(raw_id)

                        count += 1
                        name = f"mol_{count}"

                        out_smi.write(f"{smi} {name}\n")
                        map_file.write(f"{name},{source},{sid}\n")

                    except Exception as e:
                        print(f"[ERROR CSV] {e}", file=sys.stderr)
                        fail += 1

        else:
            raise ValueError(f"Unsupported file format: {ext}")

# ============================================================
# FINAL
# ============================================================

except Exception as e:
    print(f"[FATAL ERROR] {e}", file=sys.stderr)
    sys.exit(1)

print("================================")
print(f"Processed: {count}")
print(f"Failed: {fail}")
print("================================")

if count == 0:
    sys.exit(2)
```

**protonate.sh:**

```
#!/usr/bin/env bash
set -euo pipefail

INPUT_SMI="$1"
OUT_BASE="$2"
PH="$3"
JOBS="$4"

# ----------------------------
# DIRECTORIES
```

```
# ----------------------------
SPLIT_DIR="$OUT_BASE/chunks"
OUT_DIR="$OUT_BASE/protonated_chunks"
LOG_DIR="$OUT_BASE/logs"
FINAL_FILE="$OUT_BASE/protonated_master.smi"
NEXT_INPUT="$OUT_BASE/protonated_smi"

rm -rf "$NEXT_INPUT"
mkdir -p "$SPLIT_DIR" "$OUT_DIR" "$LOG_DIR" "$NEXT_INPUT"

echo "[INFO] Protonation started"
echo "[INFO] Input: $INPUT_SMI"
echo "[INFO] pH: $PH"
echo "[INFO] Jobs: $JOBS"

# ----------------------------
# CHECK INPUT
# ----------------------------
if [[ ! -s "$INPUT_SMI" ]]; then
    echo "[ERROR] Empty or missing input SMILES"
    exit 1
fi

# ----------------------------
# SPLIT INPUT
# ----------------------------
split -n l/$JOBS "$INPUT_SMI" "$SPLIT_DIR/chunk_"

echo "[INFO] Total ligands:"
wc -l "$INPUT_SMI"

# ----------------------------
# PROTONATION (PARALLEL)
# ----------------------------
echo "[INFO] Running Dimorphite-DL..."

find "$SPLIT_DIR" -name "chunk_*" | parallel -j "$JOBS" '
chunk={}
base=$(basename "$chunk")

outfile='"$OUT_DIR"'/${base}_pH.smi
logfile='"$LOG_DIR"'/${base}.log
tmpfile='"$OUT_DIR"'/${base}_tmp.smi

dimorphite_dl "$chunk" \
    --ph_min '"$PH"' \
```

```
    --ph_max '"$PH"' \
    --max_variants 1 \
    > "$tmpfile" 2>> "$logfile"

paste -d " " "$tmpfile" <(awk "{print \$2}" "$chunk") > "$outfile"
rm -f "$tmpfile"
'

# ----------------------------
# MERGE
# ----------------------------
cat "$OUT_DIR"/*_pH.smi | sed '/^$/d' > "$FINAL_FILE"

echo "[INFO] Protonated molecules:"
wc -l "$FINAL_FILE"

# ----------------------------
# FIXED SPLITTING (IMPORTANT FIX)
# ----------------------------
echo "[INFO] Creating per-ligand SMILES..."

while read -r smi name; do
    [[ -z "$smi" || -z "$name" ]] && continue
    echo "$smi $name" > "$NEXT_INPUT/${name}.smi"
done < "$FINAL_FILE"

echo "[INFO] Generated files:"
ls "$NEXT_INPUT" | wc -l

echo "[SUCCESS] Protonation complete"
```

**generate_3d.py:**

```
#!/usr/bin/env python3

import argparse
import os
import sys
from rdkit import Chem
from rdkit.Chem import AllChem, Descriptors

# ==============================================================
# CLI
# ==============================================================
```

```
parser = argparse.ArgumentParser(description="Generate 3D conformers
from SMILES")
parser.add_argument("--input", required=True, help="Input .smi file
(single ligand)")
parser.add_argument("--outdir", required=True, help="Output directory
for SDF")

parser.add_argument("--nconf", type=int, default=12)
parser.add_argument("--keep", type=int, default=1)
parser.add_argument("--maxiters", type=int, default=500)
parser.add_argument("--seed", type=int, default=42)

args = parser.parse_args()

# ============================================================
# Validate input (IMPORTANT FIX)
# ============================================================
if not os.path.exists(args.input):
    print(f"[ERROR] Input file not found: {args.input}")
    sys.exit(1)

if os.path.getsize(args.input) == 0:
    print(f"[ERROR] Input file is empty: {args.input}")
    sys.exit(1)

# ============================================================
# Setup output
# ============================================================
os.makedirs(args.outdir, exist_ok=True)

infile = args.input
base = os.path.basename(infile).replace(".smi", "")
outfile = os.path.join(args.outdir, f"{base}_3D.sdf")

# ============================================================
# Main execution
# ============================================================
try:
    print("====================================")
    print("Ligand:", infile)

    # ------------------------------
    # READ SMILES
    # ------------------------------
    with open(infile) as f:
        line = f.readline().strip()
```

```
if not line:
    raise ValueError("Empty SMILES file")

parts = line.split()
smiles = parts[0]
name = parts[1] if len(parts) > 1 else base

# ------------------------------
# MOLECULE CREATION
# ------------------------------
mol = Chem.MolFromSmiles(smiles)

if mol is None:
    raise ValueError(f"Invalid SMILES: {smiles}")

mol.SetProp("_Name", name)

# IMPORTANT: hydrogens added AFTER protonation step
mol = Chem.AddHs(mol)

print("Atoms:", mol.GetNumAtoms())
print("Rotatable bonds:", Descriptors.NumRotatableBonds(mol))

# ============================================================
# CONFORMER GENERATION
# ============================================================
params = AllChem.ETKDGv3()
params.randomSeed = args.seed
params.pruneRmsThresh = 0.5
params.useSmallRingTorsions = True
params.numThreads = 1

conf_ids = AllChem.EmbedMultipleConfs(
    mol,
    numConfs=args.nconf,
    params=params
)

print("Conformers generated:", len(conf_ids))

if len(conf_ids) == 0:
    raise ValueError("No conformers generated")

energies = []
```

```
# ============================================================
# ENERGY OPTIMIZATION
# ============================================================
if AllChem.MMFFHasAllMoleculeParams(mol):
    print("Force field: MMFF")

    results = AllChem.MMFFOptimizeMoleculeConfs(
        mol,
        maxIters=args.maxiters
    )

    for cid, (not_conv, energy) in enumerate(results):
        energies.append((cid, energy, not_conv))

else:
    print("Force field: UFF")

    for cid in conf_ids:
        not_conv = AllChem.UFFOptimizeMolecule(
            mol,
            confId=cid,
            maxIters=args.maxiters
        )
        ff = AllChem.UFFGetMoleculeForceField(mol, confId=cid)
        energy = ff.CalcEnergy()
        energies.append((cid, energy, not_conv))

# ============================================================
# SORT CONFORMERS
# ============================================================
energies.sort(key=lambda x: x[1])

print("Conformer energies:")
for cid, energy, nc in energies:
    status = "OK" if nc == 0 else "NOT_CONVERGED"
    print(f"conf {cid} : {energy:.4f} ({status})")

# ============================================================
# SELECT BEST CONFORMERS
# ============================================================
converged = [e for e in energies if e[2] == 0]

if converged:
    print("Using only converged conformers")
    selected = converged
else:
```

```
        print("WARNING: No converged conformers, using all")
        selected = energies

    # ============================================================
    # WRITE OUTPUT
    # ============================================================
    writer = Chem.SDWriter(outfile)

    written = 0

    for cid, energy, nc in selected[:args.keep]:

        mol.SetProp("Energy", str(energy))
        mol.SetProp("Converged", "yes" if nc == 0 else "no")
        mol.SetProp(
            "Method",
            "MMFF" if AllChem.MMFFHasAllMoleculeParams(mol) else "UFF"
        )

        writer.write(mol, confId=cid)
        written += 1

    writer.close()

    if written == 0:
        raise ValueError("No conformers written to output")

    print("SUCCESS")
    sys.exit(0)

# ============================================================
# ERROR HANDLING
# ============================================================
except Exception as e:
    print("FAILED:", str(e))
    sys.exit(1)
```
