# Supplementary material for: An extension of jamdock-suite for virtual screening from raw chemical datasets
Source: STAR Protoc. 2026 Sep 7;7(3):104822. doi: 10.1016/j.xpro.2026.104822 (PMC13615501; doi:10.1016/j.xpro.2026.104822)
Supplement: Supplementary file 3 [file mmc3.pdf]

Data S3. Code for the jamreceptor script

jamreceptor:

```
#!/bin/bash
# Author: José Antonio Manso García
# License: CC BY-NC 4.0
# This script is licensed under the Creative Commons Attribution-
NonCommercial 4.0 International License.
# It may not be used for commercial purposes without explicit
permission from the author.
# More info: https://creativecommons.org/licenses/by-nc/4.0/

# Configure environment variables

if [[ "$OSTYPE" == "darwin"* ]]; then
	# macOS
		export PATH=$HOME/Programs/mgltools_1.5.7_MacOS-X/bin:$PATH
		export PATH=$HOME/Programs/mgltools_1.5.7_MacOS-
X/MGLToolsPckgs/AutoDockTools/Utilities24:$PATH
	else
	# Linux
		export
PATH=$HOME/Programs/mgltools_x86_64Linux2_1.5.7/bin:$PATH
		export
PATH=$HOME/Programs/mgltools_x86_64Linux2_1.5.7/MGLToolsPckgs/AutoDock
Tools/Utilities24:$PATH
fi

# Input file
read -p "Enter the name of the PDB file to process (e.g., 2q5k.pdb): "
INPUT_PDB

# Trim whitespace
INPUT_PDB="$(echo "$INPUT_PDB" | xargs)"

# Check if file exists
if [[ ! -f "$INPUT_PDB" ]]; then
    echo "Error: File '$INPUT_PDB' not found."
    exit 1
fi

# Selection of chain ID(s)
```

```
read -p "Enter the chain ID(s) to analyze (e.g., A or A B): "
SELECTED_CHAINS
SELECTED_CHAINS="$(echo "$SELECTED_CHAINS" | xargs)"

# Set cleaned file name
CLEANED_PDB="${INPUT_PDB%.*}_cleaned.pdb"

echo "Cleaning input PDB: keeping chains [$SELECTED_CHAINS], removing
waters, ligands, and other chains..."

# Cleaning the PDB file. Keep only ATOM lines from selected chains and
standard amino acids
awk -v chains="$SELECTED_CHAINS" '
    function maybe_output(line,   resn, alt, chain) {
        resn = substr(line,18,3)
        alt = substr(line,17,1)
        chain = substr(line,22,1)

        if ((alt == " " || alt == "A") &&
            keep_chain[chain] &&
            resn in amino_acids) {
            print substr(line,1,16) " " substr(line,18)  # Clear
ALTLOC
        }
    }

    BEGIN {
        n = split(chains, cl, /[[:space:]]+/)
        for (i = 1; i <= n; i++) keep_chain[cl[i]] = 1

        # Standard 20 amino acids
        amino_acids["ALA"]
        amino_acids["ARG"]
        amino_acids["ASN"]
        amino_acids["ASP"]
        amino_acids["CYS"]
        amino_acids["GLU"]
        amino_acids["GLN"]
        amino_acids["GLY"]
        amino_acids["HIS"]
        amino_acids["ILE"]
        amino_acids["LEU"]
        amino_acids["LYS"]
        amino_acids["MET"]
        amino_acids["PHE"]
        amino_acids["PRO"]
```

```
        amino_acids["SER"]
        amino_acids["THR"]
        amino_acids["TRP"]
        amino_acids["TYR"]
        amino_acids["VAL"]
    }

    /^ATOM/ {
        maybe_output($0)
    }
' "$INPUT_PDB" > "$CLEANED_PDB"

# Validate cleaned PDB
if [[ ! -s "$CLEANED_PDB" || ! $(grep "^ATOM" "$CLEANED_PDB") ]]; then
    echo "Error: The cleaned PDB file '$CLEANED_PDB' does not contain
any valid atoms."
    exit 1
fi

echo "Cleaned PDB saved as '$CLEANED_PDB'."

# Extraction the base name
BASENAME="${INPUT_PDB%.*}"
OUTPUT_PDBQT="${BASENAME}.pdbqt"
LOGFILE="${BASENAME}_prep.log"

# Searching for prepare_receptor4.py in the home directory (update if
MGLtools is not installed in the home directory)
echo "Searching for prepare_receptor4.py under your home directory..."
PREP_SCRIPT=$(find ~ -path
"*AutoDockTools*/Utilities24/prepare_receptor4.py" 2>/dev/null | head
-n 1)

if [[ -z "$PREP_SCRIPT" ]]; then
    echo "Error: Could not find prepare_receptor4.py in your home
directory."
    echo "Please check if MGLTools is installed correctly."
    exit 1
else
    echo "Found prepare_receptor4.py at: $PREP_SCRIPT"
fi

# Check if pythonsh exists
if ! command -v pythonsh &> /dev/null; then
    echo "Error: pythonsh (from MGLTools) not found in PATH."
```

```
    echo "Try adding it with: export
PATH=\$PATH:~/path/to/MGLTools/bin"
    exit 1
fi

# Run preparation scripts
echo "Running receptor preparation (PDB to PDBQT)..."
pythonsh "$PREP_SCRIPT" \
    -r "$CLEANED_PDB" \
    -o "$OUTPUT_PDBQT" \
    -A hydrogens \
    -U nphs_lps_waters_nonstdres \
    -v \
    | tee "$LOGFILE"

echo "Done! Output saved to '$OUTPUT_PDBQT'. Log written to
'$LOGFILE'."

# Run Fpocket
echo "Running Fpocket..."

      # Prepare output directory
      OUT_DIR="${BASENAME}_out"
      POCKET_INFO="${OUT_DIR}/${BASENAME}_info.txt"
      POCKET_DIR="${OUT_DIR}/pockets"

      # Create output directory if it doesn't exist
      if [[ ! -d "$OUT_DIR" ]]; then
            mkdir -p "$OUT_DIR"
      fi

      fpocket -f "$OUTPUT_PDBQT" -o "$OUT_DIR"

      # Check if pocket information file exists
      if [[ ! -f "$POCKET_INFO" ]]; then
            echo "Error: Pocket info not found. Fpocket may have
failed."
            exit 1
      fi

echo ""
echo "Detected pockets:"
echo "-----------------"

# Parse pocket information to identify potential druggable pockets
pocket_number=""
```

```
while IFS= read -r line; do
    if [[ "$line" =~ ^Pocket\ ([0-9]+) ]]; then
        pocket_number=${BASH_REMATCH[1]}
    elif [[ "$line" =~ Druggability\
Score[[:space:]]*:[[:space:]]*([0-9]+\.[0-9]+) ]]; then
        drug_score=${BASH_REMATCH[1]}
        if (( $(echo "$drug_score > 0.15" | bc -l) )); then
            echo "Pocket $pocket_number (could be druggable)"
        else
            echo "Pocket $pocket_number"
        fi
    fi
done < "$POCKET_INFO"

echo ""
echo "To view the detected pockets in Pymol:"
echo "- Press Ctrl + C to exit this script, then open
'${OUT_DIR}/${BASENAME}.pml' using PyMOL."
echo ""
read -p "Enter the pocket number(s) to use (separated by spaces): "
SELECTED_POCKETS

# Process selected pockets
coords=""
for ID in $SELECTED_POCKETS; do
    POCKET_PDB="$POCKET_DIR/pocket${ID}_atm.pdb"
    if [[ -f "$POCKET_PDB" ]]; then
        coords+=$(awk '/^ATOM/ {
            x = substr($0, 31, 8) + 0
            y = substr($0, 39, 8) + 0
            z = substr($0, 47, 8) + 0
            printf "%.3f %.3f %.3f\n", x, y, z
        }' "$POCKET_PDB")
        coords+=$'\n'
    else
        echo "Warning: Pocket $ID not found, skipping."
    fi
done

# Removes empty lines and check coordinates are present
coords=$(echo "$coords" | grep -v '^$')
if [[ -z "$coords" ]]; then
    echo "Error: No coordinates found in selected pockets."
    exit 1
fi
```

```
# Initialize grid box values with the first coordinate
read x y z <<< "$(echo "$coords" | head -n 1)"
xmin=$x; xmax=$x
ymin=$y; ymax=$y
zmin=$z; zmax=$z

# Calculate limit values
while read x y z; do
    [[ -z "$x" || -z "$y" || -z "$z" ]] && continue
    (( $(echo "$x < $xmin" | bc -l) )) && xmin=$x
    (( $(echo "$x > $xmax" | bc -l) )) && xmax=$x
    (( $(echo "$y < $ymin" | bc -l) )) && ymin=$y
    (( $(echo "$y > $ymax" | bc -l) )) && ymax=$y
    (( $(echo "$z < $zmin" | bc -l) )) && zmin=$z
    (( $(echo "$z > $zmax" | bc -l) )) && zmax=$z
done <<< "$coords"

# Show limit values
echo "xmin=$xmin, xmax=$xmax"
echo "ymin=$ymin, ymax=$ymax"
echo "zmin=$zmin, zmax=$zmax"

# Ask for entering a padding
read -p "Enter padding size in Ångströms (default 10.0): " PADDING
PADDING=${PADDING:-10.0}
echo "Using padding: $PADDING Å"

# Calculate center and size
center_x=$(echo "($xmin + $xmax) / 2" | bc -l)
center_y=$(echo "($ymin + $ymax) / 2" | bc -l)
center_z=$(echo "($zmin + $zmax) / 2" | bc -l)

size_x=$(echo "$xmax - $xmin + $PADDING" | bc -l)
size_y=$(echo "$ymax - $ymin + $PADDING" | bc -l)
size_z=$(echo "$zmax - $zmin + $PADDING" | bc -l)

# Save configuration file
cat <<EOF > conf.txt
receptor = $(printf "%s"  "$BASENAME.pdbqt")
center_x = $(printf "%.1f" "$center_x")
center_y = $(printf "%.1f" "$center_y")
center_z = $(printf "%.1f" "$center_z")
size_x   = $(printf "%.0f" "$size_x")
size_y   = $(printf "%.0f" "$size_y")
size_z   = $(printf "%.0f" "$size_z")
cpu = 1
```

```
EOF

echo "Grid box parameters written to 'conf.txt'."

# Generate grid_box.py
GRID_PY="grid_box.py"

cat <<EOF > "$GRID_PY"
from pymol import cmd
from pymol.cgo import *

# Define grid box dimensions
center_x = $(printf "%.1f" "$center_x")
center_y = $(printf "%.1f" "$center_y")
center_z = $(printf "%.1f" "$center_z")
size_x = $(printf "%.0f" "$size_x")
size_y = $(printf "%.0f" "$size_y")
size_z = $(printf "%.0f" "$size_z")

# Calculate the corners of the box
x_min = center_x - size_x / 2
x_max = center_x + size_x / 2
y_min = center_y - size_y / 2
y_max = center_y + size_y / 2
z_min = center_z - size_z / 2
z_max = center_z + size_z / 2

# Create a list for the box, properly formatted to avoid line breaks
box = [
    BEGIN, LINE_STRIP,
    VERTEX, x_min, y_min, z_min,
    VERTEX, x_max, y_min, z_min,
    VERTEX, x_max, y_max, z_min,
    VERTEX, x_min, y_max, z_min,
    VERTEX, x_min, y_min, z_min,

    VERTEX, x_min, y_min, z_max,
    VERTEX, x_max, y_min, z_max,
    VERTEX, x_max, y_max, z_max,
    VERTEX, x_min, y_max, z_max,
    VERTEX, x_min, y_min, z_max,

    VERTEX, x_min, y_min, z_min,
    VERTEX, x_min, y_min, z_max,

    VERTEX, x_max, y_min, z_min,
```

```
    VERTEX, x_max, y_min, z_max,

    VERTEX, x_max, y_max, z_min,
    VERTEX, x_max, y_max, z_max,

    VERTEX, x_min, y_max, z_min,
    VERTEX, x_min, y_max, z_max,
    END
]

# Load the grid box into PyMOL
cmd.load_cgo(box, 'grid_box')
EOF

echo "Visualization script 'grid_box.py' generated."

# Display citation message
echo ""
echo
"=================================================================="
echo " If you are using this program or its outputs, please cite:"
echo ""
echo " Manso, J.A. (2025). Zenodo,
https://doi.org/10.5281/zenodo.15577778"
echo ""
echo " Morris et al. (2009). J Comput Chem 30, 2785–2791."
echo " https://doi.org/10.1002/jcc.21256"
echo ""
echo " Le Guilloux et al (2009). BMC Bioinformatics 10, 168."
echo " https://doi.org/10.1186/1471-2105-10-168"
echo
"=================================================================="
```
