# Supplementary material for: An extension of jamdock-suite for virtual screening from raw chemical datasets
Source: STAR Protoc. 2026 Sep 7;7(3):104822. doi: 10.1016/j.xpro.2026.104822 (PMC13615501; doi:10.1016/j.xpro.2026.104822)
Supplement: Supplementary file 4 [file mmc4.pdf]

Data S4. Code for the sem_dock script

sem_dock:

```
#!/usr/bin/env bash
set -euo pipefail

# ============================================================
# SEM_DOCK — High-Throughput Docking
# ============================================================

CPU="8"
CPU_FRACTION=0.9
LIG_DIR="../prep_out"
OUTDIR="."
CONFIG="conf.txt"

# Defaults (used only if not provided anywhere)
DEFAULT_EXHAUSTIVENESS=8
DEFAULT_MAX_POSES=9
DEFAULT_ENERGY_RANGE=3

# User-provided (optional)
EXHAUSTIVENESS=""
MAX_POSES=""
ENERGY_RANGE=""

usage() {
cat << EOF

============================================================
 SEM_DOCK — High-Throughput Docking (AutoDock Vina)
============================================================

USAGE:
  sem_dock --config conf.txt --lig_dir prep_out [options]

OPTIONS:
  --cpu INT
  --cpu_fraction FLOAT
  --exhaustiveness INT
  --max_poses INT
  --energy_range FLOAT
  --out_dir DIR
```

```
Priority:
  CLI > config file > default

EOF
exit 1
}
[[ $# -eq 0 ]] && usage

# ----------------------------
# Parse args
# ----------------------------
while [[ $# -gt 0 ]]; do
    case $1 in
        --config) CONFIG="$2"; shift 2 ;;
        --lig_dir) LIG_DIR="$2"; shift 2 ;;
        --cpu) CPU="$2"; shift 2 ;;
        --cpu_fraction) CPU_FRACTION="$2"; shift 2 ;;
        --exhaustiveness) EXHAUSTIVENESS="$2"; shift 2 ;;
        --max_poses) MAX_POSES="$2"; shift 2 ;;
        --energy_range) ENERGY_RANGE="$2"; shift 2 ;;
        --out_dir) OUTDIR="$2"; shift 2 ;;
        -h|--help) usage ;;
        *) usage ;;
    esac
done

[[ -z "$CONFIG" || -z "$LIG_DIR" ]] && usage

# ----------------------------
# Helper: read config
# ----------------------------
get_config_value() {
    grep -E "^$1\s*=" "$CONFIG" | awk -F= '{print $2}' | xargs || true
}

CONF_EXH=$(get_config_value "exhaustiveness")
CONF_MODES=$(get_config_value "num_modes")
CONF_RANGE=$(get_config_value "energy_range")

# ----------------------------
# Resolve final values
# Priority: CLI > config > default
# ----------------------------
EXHAUSTIVENESS="${EXHAUSTIVENESS:-${CONF_EXH:-
$DEFAULT_EXHAUSTIVENESS}}"
MAX_POSES="${MAX_POSES:-${CONF_MODES:-$DEFAULT_MAX_POSES}}"
```

```
ENERGY_RANGE="${ENERGY_RANGE:-${CONF_RANGE:-$DEFAULT_ENERGY_RANGE}}"

# ----------------------------
# Validate
# ----------------------------
[[ "$EXHAUSTIVENESS" =~ ^[0-9]+$ ]] || { echo "[ERROR] Invalid
exhaustiveness"; exit 1; }
[[ "$MAX_POSES" =~ ^[0-9]+$ ]] || { echo "[ERROR] Invalid max_poses";
exit 1; }
[[ "$ENERGY_RANGE" =~ ^[0-9]+(\.[0-9]+)?$ ]] || { echo "[ERROR]
Invalid energy_range"; exit 1; }

# ----------------------------
# Normalize paths
# ----------------------------
PDBQT_DIR="$LIG_DIR/pdbqt"

[[ ! -d "$PDBQT_DIR" ]] && { echo "[ERROR] Ligand dir not found:
$PDBQT_DIR"; exit 1; }
[[ ! -f "$CONFIG" ]] && { echo "[ERROR] Config not found: $CONFIG";
exit 1; }

# ----------------------------
# Output dir
# ----------------------------
if [[ -z "$OUTDIR" ]]; then
    timestamp=$(date +"%Y%m%d_%H%M%S")
    OUTDIR="dock_${timestamp}"
fi

OUT_DIR="$OUTDIR/vina_out"
LOG_DIR="$OUTDIR/vina_logs"

mkdir -p "$OUT_DIR" "$LOG_DIR"

# ----------------------------
# Show config summary
# ----------------------------
echo "[INFO] Exhaustiveness: $EXHAUSTIVENESS"
echo "[INFO] Max poses: $MAX_POSES"
echo "[INFO] Energy range: $ENERGY_RANGE"

# ----------------------------
# CPU handling
# ----------------------------
TOTAL_CPU=$(nproc)
```

```
if [[ -n "${CPU:-}" ]]; then
    JOBS="$CPU"
else
    JOBS=$(python3 - <<EOF
import math
print(max(1, int($TOTAL_CPU * $CPU_FRACTION)))
EOF
)
fi

echo "[INFO] CPUs: $TOTAL_CPU | Using: $JOBS"

# ----------------------------
# Ligand list
# ----------------------------
LIG_LIST="$OUTDIR/ligands.txt"
find "$PDBQT_DIR" -maxdepth 1 -type f -name "*.pdbqt" | sort -V >
"$LIG_LIST"

TOTAL=$(wc -l < "$LIG_LIST")
[[ "$TOTAL" -eq 0 ]] && { echo "[ERROR] No ligands found"; exit 1; }

echo "[INFO] Total ligands: $TOTAL"

# ----------------------------
# Progress tracking
# ----------------------------
COUNTER_FILE="$OUTDIR/progress.txt"
LOCK_FILE="$COUNTER_FILE.lock"
START_FILE="$OUTDIR/start_time.txt"

echo 0 > "$COUNTER_FILE"
date +%s > "$START_FILE"

START_TIME=$(cat "$START_FILE")

# ----------------------------
# Run docking
# ----------------------------
echo "[INFO] Starting docking..."

export OUT_DIR LOG_DIR CONFIG COUNTER_FILE TOTAL LOCK_FILE START_TIME
\
       EXHAUSTIVENESS MAX_POSES ENERGY_RANGE OUTDIR
```

```
cat "$LIG_LIST" | parallel -j "$JOBS" --halt never '
lig={}
base=$(basename "$lig" .pdbqt)

out="$OUT_DIR/${base}_out.pdbqt"
log="$LOG_DIR/${base}.log"

[[ -s "$out" ]] && exit 0

# Build Vina command dynamically
cmd=(vina --config "$CONFIG" --cpu 1 --ligand "$lig" --out "$out")

[[ -n "$EXHAUSTIVENESS" ]] && cmd+=(--exhaustiveness
"$EXHAUSTIVENESS")
[[ -n "$MAX_POSES" ]] && cmd+=(--num_modes "$MAX_POSES")
[[ -n "$ENERGY_RANGE" ]] && cmd+=(--energy_range "$ENERGY_RANGE")

"${cmd[@]}" > "$log" 2>&1

if [[ ! -s "$out" ]]; then
    echo "$base" >> "$OUTDIR/failed_docking.txt"
    exit 0
fi

(
    flock 200

    count=$(cat "$COUNTER_FILE")
    count=$((count + 1))
    echo "$count" > "$COUNTER_FILE"

    now=$(date +%s)
    elapsed=$((now - START_TIME))

    if [[ "$elapsed" -gt 0 ]]; then
        rate=$(awk "BEGIN {print $count/$elapsed}")
        remaining=$((TOTAL - count))
        eta_sec=$(awk "BEGIN {print $remaining/$rate}")
        finish_epoch=$(awk "BEGIN {print int($now + $eta_sec)}")

        if awk "BEGIN {exit !($eta_sec > 86400)}"; then
            finish_time=$(date -d "@$finish_epoch" "+%Y-%m-%d %H:%M")
        else
            finish_time=$(date -d "@$finish_epoch" "+%H:%M:%S")
        fi
```

```
        per_min=$(awk "BEGIN {print $rate*60}")
        eta_min=$(awk "BEGIN {print $eta_sec/60}")
    else
        per_min=0
        eta_min=0
        finish_time="..."
    fi

    printf "\r[%-6d/%-6d] %6.2f lig/min | ETA: %6.1f min | Finish: %s"
\
    "$count" "$TOTAL" "$per_min" "$eta_min" "$finish_time"

) 200>"$LOCK_FILE"
'

echo ""


# ----------------------------
# Cleanup
# ----------------------------
rm -f "$COUNTER_FILE" "$LOCK_FILE" "$START_FILE"

echo "[SUCCESS] Docking completed"
```
