# Supplementary material for: An extension of jamdock-suite for virtual screening from raw chemical datasets
Source: STAR Protoc. 2026 Sep 7;7(3):104822. doi: 10.1016/j.xpro.2026.104822 (PMC13615501; doi:10.1016/j.xpro.2026.104822)
Supplement: Supplementary file 5 [file mmc5.pdf]

Data S5. Code for the sem_filter script

sem_filter:

```
#!/usr/bin/env python3

import os
import sys
import csv
import argparse
from rdkit import Chem
from rdkit.Chem import Descriptors, Crippen

# ============================================================
# HELP MESSAGE
# ============================================================

def print_help():
    print(r"""
============================================================
 SEM_FILTER – Docking Results Analysis & Filtering
============================================================

DESCRIPTION:
  Analyzes AutoDock Vina docking results and generates:

    • Results.csv (ALL ligands)
    • Filtered_Results.csv (filtered subset)
    • Filtered_Features.csv (RDKit descriptors)

  Optional:
    • SimScore calculation (--simscore)

------------------------------------------------------------
USAGE:
  sem_filter --dock_dir DIR --prep_dir DIR [options]

------------------------------------------------------------
REQUIRED:
  --dock_dir DIR
  --prep_dir DIR

------------------------------------------------------------
OPTIONAL:
  --threshold FLOAT     Affinity cutoff (e.g. -7.0)
```

```
  --top INT              Select top N ligands
  --simscore             Enable SimScore calculation
  --out_dir DIR           Output directory

------------------------------------------------------------
EXAMPLES:

  sem_filter --dock_dir dock_run --prep_dir prep_out

  sem_filter --dock_dir dock_run --prep_dir prep_out --simscore

  sem_filter --dock_dir dock_run --prep_dir prep_out --top 100 --
simscore

============================================================
""")

# ============================================================
# CLI
# ============================================================

parser = argparse.ArgumentParser(add_help=False)

parser.add_argument("--dock_dir")
parser.add_argument("--prep_dir")
parser.add_argument("--threshold", type=float, default=None)
parser.add_argument("--top", type=int, default=None)
parser.add_argument("--simscore", action="store_true")
parser.add_argument("--out_dir", default=None)
parser.add_argument("-h", "--help", action="store_true")

args = parser.parse_args()

if args.help or len(sys.argv) == 1:
    print_help()
    sys.exit(0)

if not args.dock_dir or not args.prep_dir:
    print("[ERROR] Missing required arguments\n")
    print_help()
    sys.exit(1)

DOCK_DIR = args.dock_dir
PREP_DIR = args.prep_dir

# ============================================================
```

```
# OUTPUT DIR
# ============================================================

ANALYSIS_DIR = args.out_dir if args.out_dir else
os.path.join(DOCK_DIR, "analysis")
os.makedirs(ANALYSIS_DIR, exist_ok=True)

print(f"[INFO] Dock dir   : {DOCK_DIR}")
print(f"[INFO] Prep dir   : {PREP_DIR}")
print(f"[INFO] Output dir : {ANALYSIS_DIR}")

# ============================================================
# PATHS
# ============================================================

PDBQT_DIR = os.path.join(DOCK_DIR, "vina_out")
SDF_DIR = os.path.join(PREP_DIR, "3D")
ID_MAP_FILE = os.path.join(PREP_DIR, "id_map.csv")

ALL_CSV = os.path.join(ANALYSIS_DIR, "Results.csv")
FILTER_CSV = os.path.join(ANALYSIS_DIR, "Filtered_Results.csv")
FEATURE_CSV = os.path.join(ANALYSIS_DIR, "Filtered_Features.csv")

# ============================================================
# LOAD ID MAP
# ============================================================

source_map = {}
id_map = {}

if os.path.exists(ID_MAP_FILE):
    with open(ID_MAP_FILE) as f:
        reader = csv.DictReader(f)
        for row in reader:
            lig = row["Ligand"].strip()
            source_map[lig] = row["Source"]
            id_map[lig] = row["SourceID"]

# ============================================================
# PARSE PDBQT
# ============================================================

def parse_pdbqt(file_path, use_simscore=False):

    affinities = []
    lb_list = []
```

```
    ub_list = []

    with open(file_path) as f:
        for line in f:
            if line.startswith("REMARK VINA RESULT:"):
                parts = line.split()
                aff = float(parts[3])
                lb = float(parts[4])
                ub = float(parts[5])

                affinities.append(aff)
                lb_list.append(lb)
                ub_list.append(ub)

    if not affinities:
        return None

    best_affinity = min(affinities)
    total_modes = len(affinities)

    simscore = None

    if use_simscore:
        if total_modes <= 1:
            simscore = 0
        else:
            count_lb = sum(1 for x in lb_list if 0 <= x < 1.6)
            count_ub = sum(1 for x in ub_list if 0 <= x < 3.2)

            pct_lb = 100 * (count_lb - 1) / total_modes
            pct_ub = 100 * (count_ub - 1) / total_modes

            simscore = round((pct_lb + pct_ub) / 2)

    return best_affinity, total_modes, simscore

# ============================================================
# COLLECT RESULTS
# ============================================================

results = []

if not os.path.exists(PDBQT_DIR):
    print(f"[ERROR] Missing vina_out directory")
    sys.exit(1)
```

```
for file in os.listdir(PDBQT_DIR):

    if not file.endswith(".pdbqt"):
        continue

    path = os.path.join(PDBQT_DIR, file)
    base = file.replace("_out.pdbqt", "").replace(".pdbqt", "")

    parsed = parse_pdbqt(path, args.simscore)

    if parsed is None:
        continue

    affinity, modes, simscore = parsed

    src = source_map.get(base, "Custom")
    sid = id_map.get(base, base)

    results.append((base, src, sid, affinity, modes, simscore))

# ============================================================
# SORT (BY AFFINITY)
# ============================================================

results.sort(key=lambda x: x[3])

# ============================================================
# FILTER
# ============================================================

filtered = results

if args.threshold is not None:
    filtered = [r for r in filtered if r[3] <= args.threshold]

if args.top is not None:
    filtered = filtered[:args.top]

# ============================================================
# WRITE RESULTS (ALL)
# ============================================================

with open(ALL_CSV, "w", newline="") as f:

    writer = csv.writer(f)
```

```
    header = ["Ligand", "Source", "SourceID", "Affinity",
"TotalModes"]

    if args.simscore:
        header.append("SimScore")

    writer.writerow(header)

    for r in results:
        row = list(r[:5])
        if args.simscore:
            row.append(r[5])
        writer.writerow(row)

# ============================================================
# WRITE FILTERED RESULTS
# ============================================================

with open(FILTER_CSV, "w", newline="") as f:

    writer = csv.writer(f)

    header = ["Ligand", "Source", "SourceID", "Affinity",
"TotalModes"]

    if args.simscore:
        header.append("SimScore")

    writer.writerow(header)

    for r in filtered:
        row = list(r[:5])
        if args.simscore:
            row.append(r[5])
        writer.writerow(row)

# ============================================================
# FEATURES (FILTERED ONLY)
# ============================================================

with open(FEATURE_CSV, "w", newline="") as f:

    writer = csv.writer(f)

    header = [
        "Ligand", "Source", "SourceID", "Affinity", "TotalModes",
```

```
        "SMILES", "MW", "TPSA", "HBD", "HBA", "RotB", "LogP"
    ]

    if args.simscore:
        header.insert(5, "SimScore")

    writer.writerow(header)

    for r in filtered:

        lig, src, sid, aff, modes, simscore = r

        sdf_file = os.path.join(SDF_DIR, f"{lig}_3D.sdf")

        base_data = [lig, src, sid, aff, modes]

        if args.simscore:
            base_data.append(simscore)

        if not os.path.exists(sdf_file):
            writer.writerow(base_data + ["NA"] * 7)
            continue

        try:
            suppl = Chem.SDMolSupplier(sdf_file, removeHs=False)
            mol = next((m for m in suppl if m is not None), None)

            if mol is None:
                writer.writerow(base_data + ["NA"] * 7)
                continue

            smiles = Chem.MolToSmiles(mol)

            desc = [
                smiles,
                f"{Descriptors.MolWt(mol):.2f}",
                f"{Descriptors.TPSA(mol):.2f}",
                Descriptors.NumHDonors(mol),
                Descriptors.NumHAcceptors(mol),
                Descriptors.NumRotatableBonds(mol),
                f"{Crippen.MolLogP(mol):.2f}",
            ]

            writer.writerow(base_data + desc)

        except:
```

```
            writer.writerow(base_data + ["NA"] * 7)

# ============================================================
# SUMMARY
# ============================================================

print("=================================")
print(f"Total ligands   : {len(results)}")
print(f"Filtered ligands: {len(filtered)}")
if args.simscore:
    print("SimScore        : Enabled")
else:
    print("SimScore        : Disabled")
print("=================================")
```
