# Supplementary material for: An extension of jamdock-suite for virtual screening from raw chemical datasets
Source: STAR Protoc. 2026 Sep 7;7(3):104822. doi: 10.1016/j.xpro.2026.104822 (PMC13615501; doi:10.1016/j.xpro.2026.104822)
Supplement: Supplementary file 6 [file mmc6.pdf]

Data S6. Code for the fail_check script

fail_check:

```
#!/usr/bin/env python3

import os
import argparse

# ============================================================
# HELP
# ============================================================

def print_help():
    print("""
============================================================
 FAIL_CHECK – Identify Failed Ligands in sem_prep
============================================================

DESCRIPTION:
  Identifies failed ligands and classifies failure stage:

    • FAILED_3D      → no conformer generated
    • FAILED_PDBQT   → conversion failure

USAGE:
  fail_check --prep_dir DIR

OUTPUT:
  prep_dir/failed_ligands.txt

============================================================
""")

# ============================================================
# ARGPARSE
# ============================================================

parser = argparse.ArgumentParser(add_help=False)
parser.add_argument("--prep_dir")
parser.add_argument("-h", "--help", action="store_true")

args = parser.parse_args()
```

```
if args.help or not args.prep_dir:
    print_help()
    exit()

PREP_DIR = args.prep_dir

# ============================================================
# LOCATE master.smi
# ============================================================

candidate_paths = [
    os.path.join(PREP_DIR, "master.smi"),
    os.path.join(PREP_DIR, "smiles", "master.smi"),
]

MASTER_SMI = None
for path in candidate_paths:
    if os.path.exists(path):
        MASTER_SMI = path
        break

if MASTER_SMI is None:
    print("[ERROR] master.smi not found")
    exit(1)

# ============================================================
# PATHS
# ============================================================

PDBQT_DIR = os.path.join(PREP_DIR, "pdbqt")
SDF_DIR = os.path.join(PREP_DIR, "3D")

OUTPUT_FILE = os.path.join(PREP_DIR, "failed_ligands.txt")

if not os.path.isdir(PDBQT_DIR):
    print(f"[ERROR] Missing: {PDBQT_DIR}")
    exit(1)

if not os.path.isdir(SDF_DIR):
    print(f"[ERROR] Missing: {SDF_DIR}")
    exit(1)

print(f"[INFO] Using master.smi: {MASTER_SMI}")

# ============================================================
# LOAD MASTER LIST
```

```
# ============================================================

all_ligands = []

with open(MASTER_SMI) as f:
    for line in f:
        line = line.strip()
        if not line:
            continue

        parts = line.split()
        if len(parts) < 2:
            continue

        lig_id = parts[-1]
        all_ligands.append(lig_id)

# ============================================================
# LOAD SUCCESS SETS
# ============================================================

pdbqt_set = set()
for f in os.listdir(PDBQT_DIR):
    if f.endswith(".pdbqt"):
        pdbqt_set.add(f.replace(".pdbqt", ""))

sdf_set = set()
for f in os.listdir(SDF_DIR):
    if f.endswith(".sdf"):
        sdf_set.add(f.replace("_3D.sdf", ""))

# ============================================================
# CLASSIFY FAILURES
# ============================================================

results = []

for lig in all_ligands:

    if lig in pdbqt_set:
        status = "OK"

    elif lig in sdf_set:
        status = "FAILED_PDBQT"

    else:
```

```
        status = "FAILED_3D"

    results.append((lig, status))

# ============================================================
# WRITE OUTPUT
# ============================================================

with open(OUTPUT_FILE, "w") as f:
    f.write("Ligand\tStatus\n")
    for lig, status in results:
        if status != "OK":
            f.write(f"{lig}\t{status}\n")

# ============================================================
# SUMMARY
# ============================================================

total = len(all_ligands)
ok = sum(1 for _, s in results if s == "OK")
fail_3d = sum(1 for _, s in results if s == "FAILED_3D")
fail_pdbqt = sum(1 for _, s in results if s == "FAILED_PDBQT")

print("================================")
print(f"Total ligands    : {total}")
print(f"Successful       : {ok}")
print(f"Failed (3D)      : {fail_3d}")
print(f"Failed (PDBQT)   : {fail_pdbqt}")
print("================================")

print(f"[INFO] Report saved: {OUTPUT_FILE}")
```
